# The axial skeleton of *Poposaurus langstoni* (Pseudosuchia: Poposauroidea) and its implications for accessory intervertebral articulation evolution in pseudosuchian archosaurs

**DOI:** 10.7717/peerj.4235

**Published:** 2018-02-14

**Authors:** Candice M. Stefanic, Sterling J. Nesbitt

**Affiliations:** Department of Geosciences, Virginia Polytechnic Institute and State University (Virginia Tech), Blacksburg, VA, USA

**Keywords:** Poposaurus, Vertebrae, Intervertebral articulation, Hyposphene–hypantrum, Body size

## Abstract

Dinosaurs and their close relatives grew to sizes larger than any other terrestrial animal in the history of life on Earth, and many enormous dinosaurs (e.g., *Diplodocus*, *Spinosaurus*, *Tyrannosaurus*) have accessory intervertebral articulations that have been suggested to support these large body sizes. Some pseudosuchian archosaurs have been reported to have these articulations as well, but few have been characterized in these taxa because of a lower abundance of complete, three-dimensional pseudosuchian vertebral material in relation to dinosaurs. We describe the axial column of the large (∼4–5 m) poposauroid pseudosuchian *Poposaurus langstoni* from the Upper Triassic of Texas (TMM Locality 31025 of the Otis Chalk localities; Dockum Group, Howard County, TX, USA). *P. langstoni* was originally named from pelvic girdle elements and vertebrae; here we describe newly discovered and prepared presacral vertebrae and a presacral rib from the original excavation of the holotype in the 1940s. The well-preserved vertebrae have well-defined vertebral laminae and clear hyposphene–hypantrum intervertebral articulations, character states mentioned in pseudosuchians but rarely described. The new material demonstrates variation present in the hyposphene–hypantrum articulation through the vertebral column. We compared these morphologies to other pseudosuchians with and without the hyposphene–hypantrum articulation. Based on these careful comparisons, we provide an explicit definition for the hyposphene–hypantrum articulation applicable across Archosauria. Within Pseudosuchia, we find the hyposphene–hypantrum appeared independently in the clade at least twice, but we also see the loss of these structures in clades that had them ancestrally. Furthermore, we found the presence of large body sizes (femoral lengths >∼300 mm) and the presence of the hyposphene–hypantrum is correlated in most non-crocodylomorph pseudosuchian archosaurs with a few exceptions. This result suggests that the presence of the hyposphene–hypantrum is controlled by the increases and decreases in body size and not strictly inheritance.

## Introduction

The clade Archosauria, which includes extant birds and crocodylians, contains some of the largest land animals ever to exist (i.e., sauropod and theropod dinosaurs, Mazzetta, Christiansen & Fariña, 2004; crocodyliforms, Sereno et al., 2001; rauisuchians, Nesbitt, Desojo & Irmis, 2013), and to understand the consequence of body size on skeletal morphologies seen only in extinct taxa, we must turn to the fossil record. The appearance of large body sizes in Archosauria during the Mesozoic Era was accompanied by large variation in vertebral morphologies (Gauthier, 1986; Apesteguia, 2005; Wilson et al., 2016). One example of a morphology present in dinosaurs that has been cited as associated with increased body size is an accessory intervertebral articulation known as the hyposphene–hypantrum articulation (Gauthier, 1986; Rauhut, 2003; Apesteguia, 2005). In addition to the centrum faces and zygapophyses, this hyposphene–hypantrum articulation forms a “lock-and-key” configuration between consecutive vertebrae. More specifically, the hyposphene–hypantrum articulation consists of a ventrally expanded lamina of bone that is present at the junction of the postzygapophyses (=the hyposphene) that fits into a complementary space (=the hypantrum) that separates the prezygapophyses at the midline (Rauhut, 2003; Apesteguia, 2005). In sauropod dinosaurs, the presence of these structures has been hypothesized to be related to increased vertebral column rigidity, thereby decreasing flexibility of locomotion (Apesteguia, 2005).

Among dinosaurs, the hyposphene–hypantrum articulation is present in many clades with exceptionally large-bodied members such as derived sauropodomorphs and theropods (Wilson, 1999; Makovicky & Norell, 2004; Brusatte & Benson, 2008; Benson, 2010; Bandyopadhyay et al., 2010; Pol, Garrido & Cerda, 2011), and it has been cited as a saurischian synapomorphy (Gauthier, 1986) because the articulation is not present in ornithischian dinosaurs. The hyposphene–hypantrum is also found to vary in shape and size relative to the rest of the neural arch in sauropods (Apesteguia, 2005). The hyposphene–hypantrum articulation, however, is not restricted to saurischian dinosaurs, and descriptions of this vertebral feature have been reported in the close relatives of dinosaurs (*Nyasasaurus parringtoni*, Nesbitt et al., 2013; *Asilisaurus kongwe*, Nesbitt et al., 2010) and in extinct archosaurs more closely related to crocodylians than to birds (Bonaparte, 1981; Azevedo, 1991; Weinbaum, 2013; Weinbaum & Hungerbühler, 2007; Parker, 2008; Nesbitt, 2005; Peyer et al., 2008; Gower & Schoch, 2009; Lautenschlager & Desojo, 2011; Nesbitt et al., 2014). Although the hyposphene–hypantrum articulation is present in some paracrocodylomorphs (e.g., *Poposaurus gracilis*, Weinbaum & Hungerbühler, 2007; *Arizonasaurus babbitti*, Nesbitt, 2005; *Postosuchus kirkpatricki*, *Postosuchus alisonae*, Peyer et al., 2008; *Prestosuchus chiniquensis*, Azevedo, 1991; *Fasolasuchus tenax*, Bonaparte, 1981; *Batrachotomus kupferzellensis*, Gower & Schoch, 2009), and in some aetosaurs (*Desmatosuchus spurensis*, Parker, 2008; *Scutarx deltatylus*, Parker, 2016b), it is not present in extant crocodylians (Romer, 1956). Previous work has not provided a comprehensive description of the hyposphene–hypantrum articulation across taxa or explored the possible homologies across archosaurs. Furthermore, the application of the term “hyposphene–hypantrum” to presacral vertebral morphology is inconsistent, and the variation of these structures, if any, is rarely reported. For each specimen described in a publication the presence or absence of a true hyposphene–hypantrum articulation was left up to the interpretation of the author, and their verdict is typically unaccompanied by an explanation.

To explore the morphology of the hyposphene–hypantrum articulation in pseudosuchians, we describe the axial column of the large pseudosuchian *Poposaurus langstoni* from the Late Triassic of Texas. The holotype specimen of this species was collected in the early 1940s near Otis Chalk, TX, USA (TMM Locality 31025) from the Dockum Group (Long & Murry, 1995). The holotype was erected solely based on the right ilium (TMM 31025-12), but additional material comprising four presacral vertebrae (eighth cervical, pathologically fused ninth cervical and first trunk, and third trunk) and a right ischium was also referred to the same species by Long & Murry (1995). Additional presacral vertebrae and most of a rib were collected from the same quarry as the holotype and previously referred material during the original excavation in the early 1940s; we attribute this material to *P. langstoni* and describe it herein for the first time because it has only been prepared recently. We also describe two vertebrae (TMM 31025-177, TMM 31025-257) in detail that were previously figured by Long & Murry (1995), but not described in detail with the previously published material. The material of *P. langstoni* we describe here is of interest because it includes a variety of presacral vertebrae (four cervical, four trunk) from throughout the column, and it is preserved in three dimensions so that articulation surfaces are easy to discern. These characteristics are not present in most Triassic pseudosuchian fossils (e.g., *Sillosuchus longicervix*, PVSJ 85, Alcober & Parrish (1997); *Ticinosuchus ferox*, PIZ T 2817, Krebs (1965)) because preservation is typically poor in Triassic-aged deposits. These vertebrae possess features that traditionally have been associated with saurischian taxa, including vertebral laminae (Wilson, 1999) and the hyposphene–hypantrum articulation (Apesteguia, 2005), and help act as a benchmark for comparison with other pseudosuchians with similar structures.

## Systematic Paleontology

ARCHOSAURIA Cope, 1869 sensu Gauthier, 1986
PSEUDOSUCHIA Zittel, 1887–1890 sensu Gauthier & Padian, 1985
POPOSAUROIDEA Nopsca, 1923 sensu Nesbitt, 2011
*POPOSAURUS LANGSTONI* Long & Murry, 1995 sensu Weinbaum & Hungerbühler, 2007

**Horizon and Locality:** Quarry 1 of the Otis Chalk localities (TMM Locality 31025), Dockum Group, Howard County, TX, USA. Biostratigraphic and lithostratigraphic correlations indicate the Otis Chalk localities are Upper Triassic (latest Carnian–early Norian) (Stocker & Kirk, 2016).

**Holotype:** TMM 31025-12, right ilium.

**Previously Referred Specimens:**

TMM 31025-257, right ischium.

TMM 31025-259, fused last cervical (ninth presacral) and first trunk (10th presacral) vertebrae.

TMM 31025-177, presacral vertebra (posterior cervical).

TMM 31025-257, presacral vertebra (anterior trunk).

**Newly Referred Specimens:**

TMM 31025-1261.5, presacral vertebra (mid-cervical).

TMM 31025-1262, presacral vertebra (mid-cervical).

TMM 31025-1261.3, presacral vertebra (mid- to posterior cervical).

TMM 31025-1261.4, presacral vertebra (anterior to mid-trunk).

TMM 31025-1261.1, presacral vertebra (anterior to mid-trunk).

TMM 31025-1261.2, presacral vertebra (mid- to posterior trunk).

TMM 31025-2160, most of a presacral rib.

**Justification for Newly Referred Material:**

**Figure 1:**
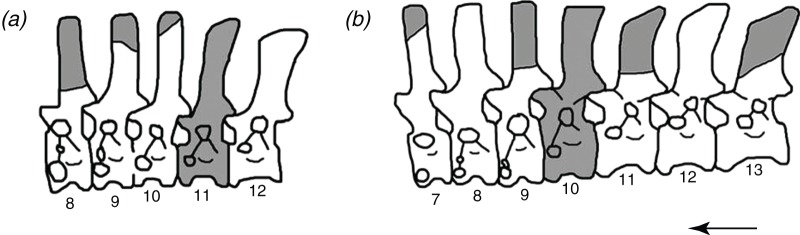
The reconstructed vertebral columns of both individuals of *Poposaurus langstoni* from the holotype locality TMM 31025, (A) individual “A” and (B) individual “B.” Material includes vertebrae described by Long & Murry (1995) and in this publication. Gray portions represent missing material. Numbers refer to presacral position within the vertebral column. Arrow indicates anterior direction. Figure not drawn to scale.

The holotype and the newly referred material were collected from the same quarry (TMM 31025 Quarry 1) during the 1940 and 1941 excavations (unpublished WPA field notes, TMM). The preservation and color of the holotype, referred, and newly referred material are identical. The relative size is also consistent between this new material and the previously referred material and holotype, but appears to represent two individuals (see below) (Fig. 1).

**Figure 2:**
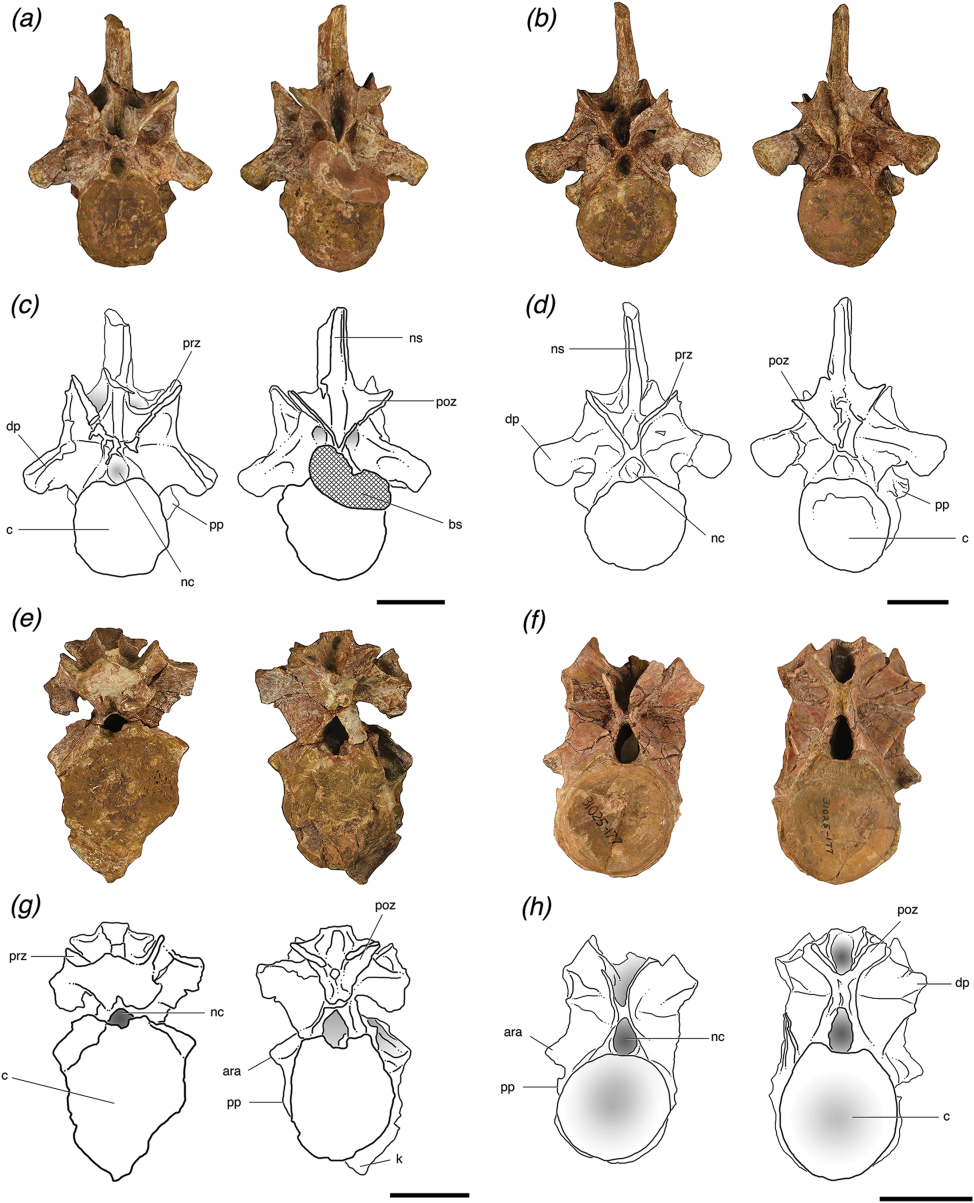
Cervical vertebrae of *Poposaurus langstoni* in anterior (left) and posterior (right) views. (A, C) TMM 31025-1261.5, presacral 7; (B, D) TMM 31025-1262, presacral 8; (E, G) TMM 31025-1261.3, presacral 9; (F, H) TMM 31025-177, presacral 8. Abbreviations: ara, accessory rib articulation; bs, bivalve shell; c, centrum; dp, diapophysis; k, keel; nc, neural canal; ns, neural spine; poz, postzygapophysis; pp, parapophysis; prz, prezygapophysis. Scales = 5 cm.

**Figure 3:**
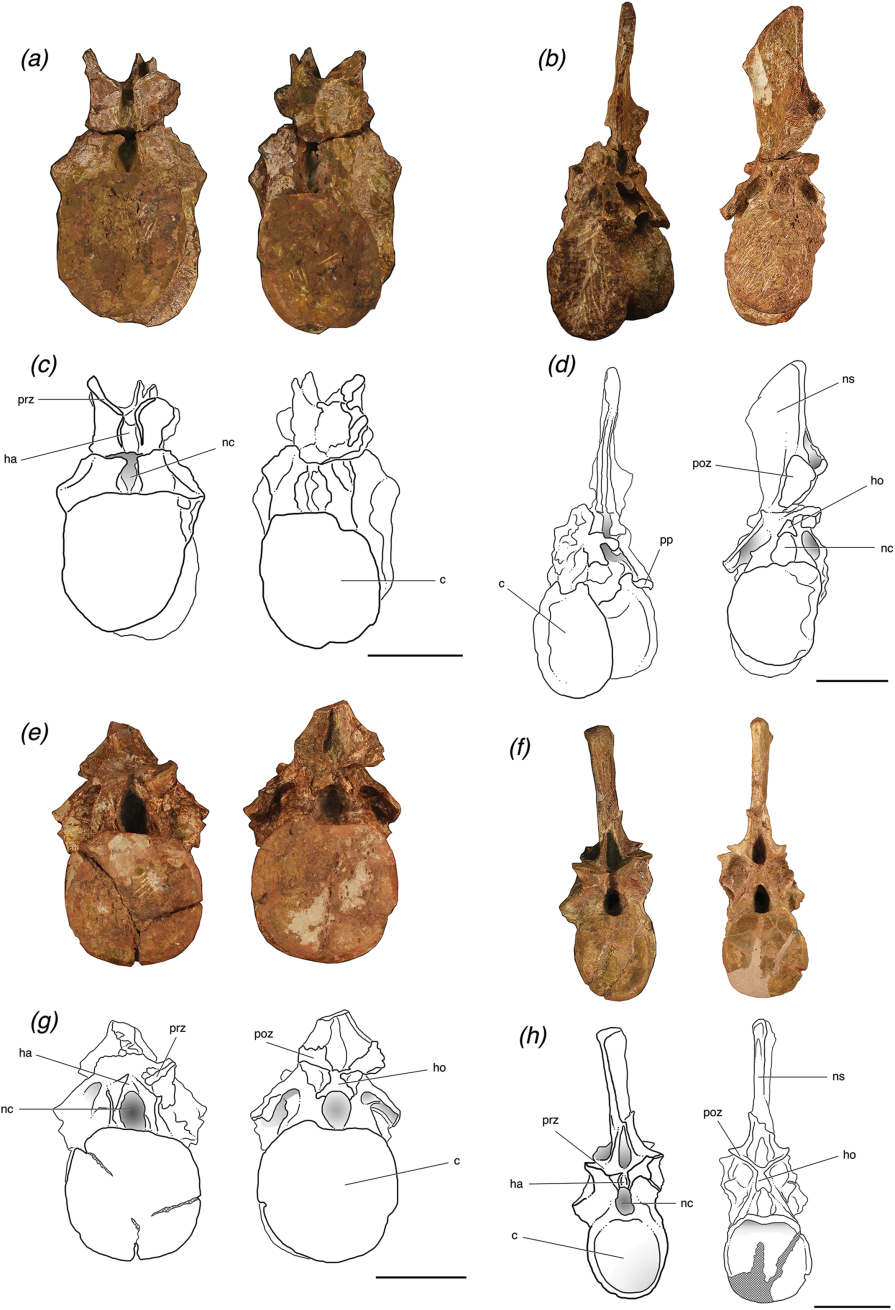
Trunk vertebrae of *Poposaurus langstoni* in anterior (left) and posterior (right) views. (A, C) TMM 31025-1261.4, presacral 11; (B, D) TMM 31025-1261.1, presacral 12; (E, G) TMM 31025-1261.2, presacral 13; (F, H) TMM 31025-257, presacral 12. Abbreviations: c, centrum; dp, diapophysis; ha, hypantrum; ho, hyposphene; nc, neural canal; ns, neural spine; poz, postzygapophysis; pp, parapophysis; prz, prezygapophysis. Scales = 5 cm.

We assign six presacral vertebrae to *P. langstoni* based on general morphology of their centra and neural arches, well-defined vertebral laminae, and an accessory rib articulation in the posterior cervical vertebrae (Figs. 2 and 3). These newly referred vertebrae share some character states with both of the previously described vertebrae of *P. langstoni* (Long & Murry, 1995; Weinbaum & Hungerbühler, 2007). The posterior cervical vertebra of the newly referred material (TMM 31025-1261.3) possesses an accessory rib articulation adjacent to the parapophyses and lacks a ridge on the ventral surface of the centum, both features that link this material to previously referred material (TMM 31025-177). This accessory rib articulation is a synapomorphy of the clade Poposauroidea (Nesbitt, 2005; Weinbaum & Hungerbühler, 2007) because it is also present in *P. gracilis* (TTU P-10419, Weinbaum & Hungerbühler, 2007), and in another member of the clade, *A. babbitti* (MSM 4590, Nesbitt, 2005). These newly referred vertebrae of *P. langstoni* also possess well-defined vertebral laminae that are also on vertebrae previously assigned to *P. gracilis* (TTU P-10419, Weinbaum & Hungerbühler, 2007), as well as on the vertebrae of many saurischian dinosaurs such as *Apatosaurus louisae* and *Allosaurus fragilis* (Wilson, 1999) and other paracrocodylomorphs such as *A. babbitti*, *Postosuchus* sp., *F. tenax*, *B. kupferzellensis*, and *P. chiniquensis* (Nesbitt, 2005; Weinbaum, 2013; Bonaparte, 1981; Gower & Schoch, 2009; Azevedo, 1991). The shape and morphologies of the centra and neural arches of the new material described here, including dorsoventrally elongate centrum facets, well-defined and pronounced laminae, and an accessory rib articulation on the cervical vertebrae, are also strikingly similar to that of *P. gracilis* (TTU P-10419, Weinbaum & Hungerbühler, 2007) and previously referred vertebrae of *P. langstoni* (TMM 31025-177, TMM 31025-257, TMM 31025-259, Long & Murry, 1995). The presence of these features in these new vertebrae allows us to assign them to the genus *Poposaurus*; however, because the diagnostic characteristics that separate *P. gracilis* and *P. langstoni* are in the ilium and ischium (Long & Murry, 1995; Weinbaum & Hungerbühler, 2007), we can only assign this material to *P. langstoni* by relying on the information that they were collected from the same quarry during the 1940 and 1941 excavations as the ilium and ischium that erected the species (unpublished WPA field notes, TMM).

We suggest that among the holotype, referred material, and newly referred material there are at least two individuals of *P. langstoni* based on a bimodal distribution of the prezygapophyseal height to centrum height, and it appears at least one of the vertebral positions is duplicated. We refer two of these vertebrae (TMM 31025-177, TMM 31025-257) to the same individual (individual “A” (Fig. 1A)) as the previously referred pathologically fused vertebrae of *P. langstoni* from Quarry 1 (TMM 31025-259) because they most closely resemble that material in size. These vertebrae (TMM 31025-177, TMM 31025-257) were both figured but not fully described by Long & Murry (1995) along with TMM 31025-259, and those authors attributed these specimens to a novel taxon, *Lythrosuchus langstoni*, because these presacral vertebrae were more compressed and taller than previously described material of *P. gracilis* and possessed an accessory rib articulation. Weinbaum & Hungerbühler (2007) later assigned these vertebrae to the same genus, *Poposaurus*, because a specimen of *P. gracilis* (TTU P-10419) also possessed the accessory rib articulation in a posterior cervical vertebra. The presacral specimens TMM 31025-1261.5, TMM 31025-1262, TMM 31025-1261.3, TMM 31025-1261.4, TMM 31025-1261.1, and TMM 31025-1261.2 we refer to as belonging to individual “B” (Fig. 1B) because they are slightly larger than those vertebrae we have assigned to individual “A.”

*Poposaurus langstoni* is only known from a single locality and other archosauromorphs are known from the same locality. The stem archosaur *Trilophosaurus buettneri* and a phytosaur are also known from TMM 31025, Quarry 1. The vertebral morphologies of the three taxa known from the locality are disparate and can easily be distinguished from one another. For example, the centra of *T. buettneri* are much longer anteroposteriorly than they are dorsoventrally tall, whereas the centra of *P. langstoni* are dorsoventrally taller than they are anteroposteriorly long. The vertebrae of *T. buettneri* (e.g., TMM 31025-140) are also much smaller in overall size than the known material of *P. langstoni* (Long & Murry, 1995), which is sufficient evidence to claim that all material from TMM 31025, Quarry 1 that is much larger than the known *T. buettneri* material is likely *P. langstoni*. These vertebrae also lack a spine table, which is evidence against them being attributed to the phytosaur taxon known from this locality. Additionally, the phytosaur material is all from a single individual and its preservation is distinct, being more yellow than this material’s brown/orange color.

## Description

### Vertebrae (general)

The axial column of *P. langstoni* consists of eight presacral vertebrae, and all are well preserved and nearly complete although most are missing parts of the diapophyses, parapophyses, zygapophyses, and some are crushed slightly (Figs. 2 and 3). Each vertebra can be assigned to a presacral position within a range of two or three positions, depending on its general position among the other vertebrae preserved and based on comparisons with other archosaurs with more complete vertebral columns. For this specimen of *P. langstoni*, all the vertebrae were assigned general positions in the vertebral column mainly based on the position of the diapophyses, as they appear higher on the neural arch further posteriorly in the series as with *P. gracilis* (TTU-P 10419), *Parringtonia gracilis* (NMT RB426), and a number of specimens of *Alligator mississippiensis* (e.g., TMM M-12606).

In *P. langstoni*, cervical vertebrae were identified by the presence of the parapophyses on the anterior rim of the centrum located anywhere from the base of the centrum to the base of the neural arch just ventral to the location of the neurocentral suture, whereas the parapophyses of trunk vertebrae are only present on the neural arch. The parapophyses migrate dorsally from the base of the centrum posteriorly along the tetrapod vertebral column (such as in *P. gracilis* and *A. mississippiensis*). Because the diapophyses are located on the neural arch in vertebrae posterior to presacral 7 in other paracrocodylomophs (e.g., *P. gracilis*, *P. gracilis*, *A. mississippiensis*) we can constrain the position of the anteriormost vertebra of *P. langstoni* (TMM 31025-1261.5) at presacral ∼7 or 8. This vertebra also has a very slight ventral keel, which further constrains its position to presacral ∼7 or 8 because this is characteristic of the most anteriorly located cervical vertebrae in other paracrocodylomorphs (e.g., *P. gracilis*).

Using these criteria for assignment of position within the axial column, the known vertebrae of *P. langstoni* include four cervical vertebrae and four trunk vertebrae (Fig. 1). All centra are amphicoelous and slightly mediolaterally compressed. All vertebrae have clearly defined vertebral laminae, and these laminae are identified using the nomenclature of Wilson (1999) and more broadly used for early archosaur taxa (Nesbitt, 2005, 2007, 2011; Nesbitt, Liu & Li, 2010; Nesbitt et al., 2014; Weinbaum, 2013; Lautenschlager & Desojo, 2011; Parker, 2008, 2016a, 2016b). All of the trunk vertebrae of this specimen preserve one or both of the accessory articulation structures (i.e., hyposphene, hypantrum) that form the hyposphene–hypantrum articulation. None of the cervical vertebrae however have the hyposphene–hypantrum present, and this is consistent with our observations that the articulation is only present in the trunk region (presacral position 10 to the last presacral before the sacrum) of archosaur vertebral columns.

### Vertebrae (cervical)

The cervical vertebra of individual “A” (TMM 31025-177) and the three cervical vertebrae of individual “B” (TMM 31025-1261.5, TMM 31025-1262, TMM 31025-1261.3) (Fig. 2) are amphicoelous, but both the posterior and anterior articular surfaces of the centrum are only slightly concave. The lateral and ventral portions of the anterior and posterior articular faces of the centra of the cervical vertebrae of individual “B” are thickened and rugose, a morphology that is more similar to *P. gracilis* than to previously described material from *P. langstoni* (Weinbaum & Hungerbühler, 2007) and the centrum of the cervical vertebra of individual “A.” The centra of these vertebrae are shorter anteroposteriorly in comparison to their dorsoventral height.

Both articular facets of the centrum of TMM 31025-1261.5 are slightly elliptical, with the long axis oriented dorsoventrally. The anterior articular facets of the centra of TMM 31025-1262, TMM 31025-1261.3, and TMM 31025-177 are circular, and the posterior articular facets of these centra are slightly elliptical with the elongated axis oriented dorsoventrally (Fig. 2). These posterior centrum facets are very similar in shape to those of the anterior cervical vertebrae of *P. kirkpatricki* (UCMP A269/124557, Long & Murry, 1995; TTU-P 9002, Weinbaum, 2013) in that they appear elliptical and elongated dorsoventrally. All of the cervical vertebrae possess deep laterally opening fossae on the centrum on both their left and right sides just dorsal to the parapophyses and about 1 cm ventral to the neurocentral sutures.

The two more posterior cervical vertebrae of individual “B” (TMM 31025-1262 and TMM 31025-1261.3) and the posterior cervical vertebra of individual “A” (TMM 31025-177) possess a well-preserved accessory rib articulation between the diapophysis and the parapophysis. The presence of these accessory articulations provides evidence that these vertebrae had double-headed ribs associated with them, an uncommon occurrence in pseudosuchians, reported only in this specimen, *P. gracilis* (TTU P-10419), and *A. babbitti* (MSM 4590). Each of the accessory rib articulations are dorsoventrally long and thin, projecting laterally from their respective centra. The accessory rib articulation of TMM 31025-1262 is broken at its base on the left lateral side, but it is intact on the right lateral side and protrudes 0.5 cm from where it attaches to the rest of the centrum. It is located 1 cm dorsal to the parapophyses. The accessory rib articulations of TMM 31025-1261.3 are intact on both sides, protrude 1.5 cm laterally, are located 3.2 cm from the diapophyses, and are located just dorsal to and touching the parapophyses. On the right lateral side of TMM 31025-177, the accessory rib articulation is just dorsal to and separated by 1 cm from the parapophyses. The left lateral side does not preserve this articulation; however, the paradiapophyseal lamina is clearly broken in the place where the articulation would be present. The accessory rib articulations on each cervical vertebra connect to the diapophyses through the paradiapophyseal laminae. This accessory rib articulation is also present in the posterior cervical vertebrae of *P. gracilis* (TTU P-10419) and *A. babbitti* (MSM 4590), and it is a synapomorphy of the clade Poposauroidea (Nesbitt, 2005; Weinbaum & Hungerbühler, 2007).

### Vertebrae (cervical; individual “A”)

#### TMM 31025-177

TMM 31025-177 is a posterior cervical vertebra. The positions of the parapophyses and diapophyses place it as likely between presacral 7 and 9, and the positions of these articulations are very similar to those in TMM 31025-1262. The parapophyses are low on the centrum and well separated from the diapophyses, which are located on the neural arch (Figs. 2F and 2H).

This specimen is mostly intact but is missing the neural spine, which is broken at the base, and the left prezygapophysis is broken. Both postzygapophyses are broken slightly at the edges and the diapophyses are both broken off where they connect to the neural arch and laminae (Figs. 2F and 2H). The neural arch is fused to the centrum, and although the neurocentral suture is nearly obliterated, there is still clear evidence of where it was located because raised areas on the lateral sides with a distinctive texture make the juncture (Brochu, 1996). The neural arch is slightly taller than the height of the centrum, and the neural arch is taller than it is laterally wide. In anterior view, the neural canal is deeply excavated and appears elliptical due to elongation of the dorsoventral axis.

TMM 30125-177 has distinct laminae forming thin, pronounced ridges on its neural arch. These laminae are thinner than those on the cervical vertebrae attributed to individual “A.” On its lateral sides there are clear posterior and anterior centrodiapophyseal laminae, which connect the diapophyses to the posterior and anterior portions of the neurocentral junction, respectively. There is a clear paradiapophyseal lamina connecting the parapophyses and diapophyses, and the accessory rib articulation is located on this lamina. This paradiapophyseal lamina extends nearly straight ventrally from the diapophysis, less than 5° from vertical. The postzygapophyseal laminae are thin and well defined and connect the parapophyses and the lateral aspect of the postzygapophyses at about a 10° angle dorsal to the anteroposterior horizontal. The prezygadiapophyseal laminae are laterally thick and short with the prezygapophyses and diapophyses less than 0.5 cm apart. These laminae are at a ∼45° angle dorsal to the anteroposterior horizontal.

### Vertebrae (cervical; individual “B”)

#### TMM 30125-1261.5

TMM 30125-1261.5 is the anteriormost vertebra of this newly referred material and can be identified as a posterior cervical vertebra (presacral 7 or 8). It is nearly complete, with only slight breakage and very minor post-depositional compression in some areas including on and just ventral to the prezygapophyses. It also has a very slight ridge along the midline of the ventral surface of the centrum. The parapophyses on the lateral sides of this vertebra are located on the base of the anterior rim of the centrum and are lowest on the centrum relative to the other vertebrae and well separated from the diapophyses. The neural arch is fused to the centrum, and the neurocentral suture is nearly obliterated, but there is a slightly raised area with a distinctive texture on the lateral surface where the suture was originally present (Brochu, 1996). In anterior view, the neural canal is circular and filled with matrix and in posterior view, the canal is obstructed by a large bivalve shell that based on general morphology, can be attributed to the freshwater mussel group Unionidae (Huber, 2010) (Figs. 2A and 2C).

The neural spine is tall and laterally compressed in TMM 31025-1261.5. A long depression extends dorsoventrally along the posterior surfaces of the neural spine. The neural spine is virtually identical in preserved morphology to that of TMM 31025-1262, but it is broken off at the dorsal end. In posterior view, the spinopostzygapophyseal laminae are paired and form distinct ridges from the base of the neural spine to nearly the distal end of the neural spine. The neural spine is flat in anterior view, and no spinoprezygapophyseal laminae are present on that side. The neural spine is much wider at its base, at 2.5 cm just dorsal to the postzygapophyses in posterior view, and tapers to about 0.5 cm thick in anterior and posterior views at its most dorsal point. It does not expand laterally into a “spine table” as in the mid-trunk vertebrae of *Nundasuchus songeaensis* (NMT RB48), the anterior and posterior trunk vertebrae of *F. tenax* (PVL 3850, PVL 3851), the trunk vertebrae of *P. chiniquensis* (UFRGS-PV-0156-T), and anterior trunk vertebrae of *B. kupferzellensis* (SMNS 80294). From lateral view, the neural spine of TMM 31025-1261.5 remains relatively consistent in anterior–posterior length at about 2.5 cm.

TMM 31025-1261.5 has distinct laminae that form thin, pronounced ridges on all surfaces of its neural arch and centrum. These laminae are more laterally expanded than the laminae of the posterior cervical vertebra of individual “B” (TMM 31025-177) and are more similar in terms of lateral expansion to the other cervical vertebrae of individual “A,” TMM 31025-1261.3 and TMM 31025-1262. On the lateral sides of TMM 31025-1261.5, there are clear anterior and posterior centrodiapophyseal laminae, which connect the diapophyses to their respective portions of the neurocentral junction. The paradiapophyseal lamina connects the parapophysis with the diapophysis, and is angled ∼5° from vertical in lateral view. The postzygapophyseal laminae are well defined and connect the parapophyses and the lateral aspect of the postzygapophyses at about a 45° angle dorsal to the anteroposterior horizontal. This specimen is crushed so that the left postzygapophysis is pushed toward the diapophysis and this has compressed the lateral side of the left postzygapophyseal lamina (Figs. 2A and 2C). The prezygadiapophyseal laminae are laterally thick and the prezygapophyses and diapophyses are ∼3 cm apart. These laminae extend dorsally from the diapophyses at a right angle to the anteroposterior horizontal with the prezygapophyses being dorsal to the diapophyses. The club-like diapophyses are fully intact where they connect to the neural arch, and the articular facets at their distal ends are elliptical. In posterior view, there are clear embayments present on the lateral sides of the transverse processes. A deep fossa is present on the left transverse process in anterior view (=prezygapophyseal centrodiapophyseal fossa of Wilson et al. (2011)), but a symmetrical counterpart is not present on the right transverse process (Figs. 2A and 2C). There are epipophyses on the dorsal portion of the postzygapophyses that form a rugose structure about 0.2 cm from the end of the articular surface. The presence of epipophyses is typically cited as a synapomorphy of Dinosauria (Long & Murry, 1995; Langer & Benton, 2006; Langer, Ezcurra & Bittencourt, 2010; Novas, 1996), but are known to occur within Pseudosuchia (Gower & Schoch, 2009; Bonaparte, 1981; Nesbitt, 2007, 2010; *Revueltosaurus callenderi*, PEFO 34561) and are found outside Archosauria among stem members (Nesbitt, 2011; Nesbitt et al., 2015). There are interzygapophyseal laminae on the dorsal portion of the prezygapophyses connecting their lateral edge to the anterior extent of the postzygapophyses, a feature also found in *Azendohsaurus madagaskarensis* (FMNH PR 3823, Nesbitt et al., 2015).

#### TMM 31025-1262

TMM 31025-1262 is a nearly complete posterior cervical vertebra, with slight breakage and minor post-depositional compression in some areas, including the prezygapophyses being pushed toward the neural spine (Figs. 2B and 2D). This specimen can be attributed to presacral 8 or 9; the parapophyses are low on the centrum, but slightly higher than those of TMM 31025-1261.5, and well separated from the diapophyses, which are located on the neural arch. TMM 31025-1262 also differs from TMM 31025-1261.5 in that it has club-like diapophyses that are more expanded and flare out at the distal end where the articular facets are located; these articular facets are circular rather than elliptical like those of TMM 31025-1261.5. The neural arch is fused to the centrum and there is some evidence of where a neurocentral suture was present on the right lateral side in the form of a small, raised ridge (Brochu, 1996). There is no keel on the ventral surface of the centrum. In anterior view, the neural canal is circular and filled with matrix, and the postzygapophyses nearly come together at the dorsal portion of the neural canal.

The neural spine is tall and mediolaterally compressed. A long depression extends dorsoventrally along the posterior surfaces of the neural spine. The neural spine is nearly identical to that of TMM 31025-1265.5. In posterior view, the spinopostzygapophyseal laminae are paired and form distinct ridges from the base to nearly the distal end of the neural spine. There are no spinoprezygapophyseal laminae present on the neural spine in anterior view. The neural spine is much wider at its base, at 3 cm dorsal to the postzygapophyses in posterior view, and tapers to about 0.5 cm mediolaterally in anterior and posterior views at its most dorsal point. It does not expand into a spine table as in the mid-trunk vertebrae of *N. songeaensis* (NMT RB48), the anterior and posterior trunk vertebrae of *F. tenax* (PVL 3850, PVL 3851), the trunk vertebrae of *P. chiniquensis* (UFRGS-PV-0156-T), and anterior trunk vertebrae of *B. kupferzellensis* (SMNS 80294). From lateral views, the neural spine remains relatively consistent in thickness mediolaterally at about 2.7 cm. The distal end of the neural spine is also rounded slightly in lateral view and is slightly taller posteriorly.

**Figure 4:**
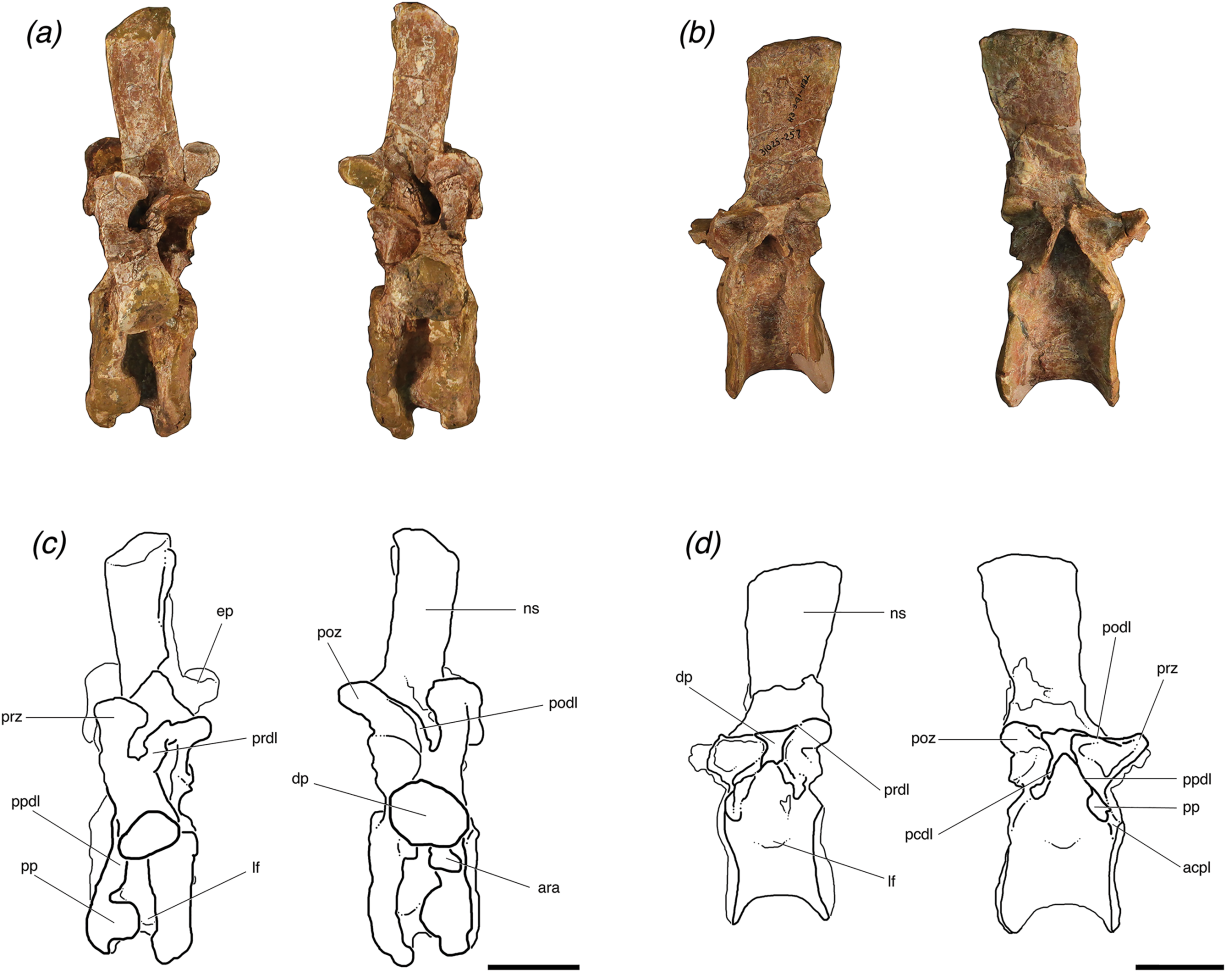
Lateral views of (A, C) one posterior cervical (TMM 31025-1262, presacral 8) and (B, D) one trunk (TMM 31025-257, presacral 12) from individuals ‘B’ and ‘A’, respectively, of *Poposaurus langstoni*. Abbreviations: acpl, anterior centroparapophyseal lamina; ara, accessory rib articulation; ep, epipophysis; dp, diapophysis; lf, lateral fossa; ns, neural spine; pcdl, posterior centrodiapophyseal lamina; podl, posteriordiapophyseal lamina; poz, postzygapophyses; pp, parapophysis; ppdl, paradiapophyseal lamina; prdl, prezygadiapophyseal lamina; prz, prezygapophysis. Scales = 5 cm.

TMM 30125-1262 has distinct laminae forming thin, pronounced ridges on its neural arch; however, these laminae are more laterally expanded than the laminae of the posterior cervical of individual “B” (TMM 31025-177) and more similar to the anterior cervical vertebrae of individual “A” (TMM 31025-1261.3 and TMM 31025-1261.5). On the lateral sides of the vertebra, there are clear posterior and anterior centrodiapophyseal laminae, which connect the diapophyses to the posterior and anterior portions of the neurocentral junction, respectively (Figs. 4A and 4C). There is a clear paradiapophyseal lamina connecting the parapophyses and diapophyses, and the accessory rib articulation is located on this lamina (Figs. 4A and 4C). This paradiapophyseal lamina extends nearly straight down from the diapophysis, ∼5° from the vertical. The postzygapophyseal laminae are well defined and connect the parapophyses and the lateral aspect of the postzygapophyses at about a 45° angle dorsal to the anteroposterior horizontal. This lamina is compressed on the left lateral side, because the specimen is crushed so that the left postzygapophysis is pushed toward the diapophysis (Figs. 2B and 2D). The prezygadiapophyseal laminae are thick in lateral view and the prezygapophyses and diapophyses are ∼3 cm apart. These laminae extend dorsally from the diapophyses at right angles to the anteroposterior horizontal plane, and the prezygapophyses are dorsal to the diapophyses. The diapophyses are intact where they connect to the neural arch, and they are more robust than in TMM 31025-1261.5 and end in circular articular facets. Centroprezygapophyseal laminae extend from the ventral side of the prezygapophyses ventrally along the neural canal to the dorsal edge of the centrum. There are epipophyses on the dorsal portion of the postzygapophyses that form a rugose structure about 0.2 cm from the end of the articular surface.

#### TMM 31025-1261.3

TMM 31025-1261.3 is a posterior cervical vertebra that can be attributed to presacral 9 or 10. The diapophyses are located on the neural arch and are well separated from the parapophyses. The parapophyses are also more dorsal on the centrum than in both TMM 31025-1261.5 and TMM 31025-1262. Although this specimen’s neural arch is detached from the centrum, the two portions were fused and then broken post-depositionally. There is evidence of where a neurocentral suture was located, because there are some slightly raised areas on the lateral sides of the centrum ventral to the break (Brochu, 1996). There is a rugose projection, which resembles a thickened and pronounced keel on the ventral surface of the centrum (Figs. 2E and 2G); however, it is asymmetrical and protrudes substantially anterior of the articular facet in anterior view. This structure seems to be pathologic in origin because it is asymmetrical and no other archosaurs possess this structure in that place.

The detached neural arch of this specimen is broken through the center of the neural canal, and is not a sufficiently close fit with the centrum to be reattached because small bits of bone are missing. The neural canal is deeply excavated; it is circular in anterior view and elliptical with the long axis oriented dorsoventrally in posterior view. The diapophyses are broken off at the point where they begin to extend out from the neural arch. A few laminae are broken and the specimen is slightly post-depositionally compressed in some areas, but otherwise it is intact.

TMM 31025-1261.3 has distinct laminae forming thin, pronounced ridges on its neural arch, and these laminae are more laterally expanded than the laminae of the cervical vertebra of individual “B” (TMM 31025-177) and more similar to the other cervical vertebrae of individual “A” (TMM 31025-1262 and TMM 31025-1261.5). On the left lateral side there is a clear posterior centrodiapophyseal laminae, which connects the diapophysis to the posterior portion of the neurocentral junction, but this lamina is broken off on the right lateral side. There is a clear paradiapophyseal lamina, which connects the parapophyses and diapophyses, but the lamina on this vertebra connects the diapophyses to the accessory rib articulation instead of the parapophyses because the articulation is located just dorsal to the parapophyses and nearly touching. The paradiapophyseal lamina extends nearly straight down from the diapophysis, ∼5° from vertical. The postzygapophyseal laminae are well defined and connect the parapophyses and the lateral aspect of the postzygapophyses at about a 45° angle dorsal to the anteroposterior horizontal. The prezygadiapophyseal laminae are thick in lateral view and the prezygapophyses and diapophyses are ∼1.5 cm apart. These laminae extend dorsally from the diapophyses at a right angle to the anteroposterior horizontal with the prezygapophyses being dorsal to the diapophyses. The diapophyses are broken off about where they connect to the neural arch, but the left one extends about 0.5 cm laterally before its break. The neural spine is broken off at its base, so spinoprezygapophyseal laminae are unable to be seen.

### Vertebrae (trunk)

The trunk vertebra of individual “A” (TMM 31025-257) and all three trunk vertebrae of individual “B” (TMM 31025-1261.4, TMM 31025-1261.1, TMM 31025-1261.2) (Fig. 3) are waisted between the articular facets of the centra. The anterior and posterior articular facets of the two anteriormost trunk vertebrae (TMM 31025-1261.4 and TMM 31025-1261.1) and the trunk vertebra of individual “A” (TMM 31025-257) appear narrow mediolaterally in lateral and ventral views. The general shape of these centra in posterior and anterior view are elliptical, with a long dorsoventrally oriented axis, which is similar to that seen in *B. kupferzellensis* (SMNS 80296, Gower & Schoch, 2009), *P. gracilis* (TTU P-10419, Weinbaum & Hungerbühler, 2007), *Effigia okeeffeae* (AMNH FR 30587, Nesbitt, 2007), and *P. kirkpatricki* (TTU P-9002, Weinbaum, 2013). The articular facets of the centrum of the most posterior trunk vertebra of individual “B” (TMM 31025-1261.2) are round and not compressed in any direction, and the anterior and posterior articular facets of the centrum appear thick (∼1 cm) in the anteroposterior direction. There are deep fossae on the centra on both the left and right lateral sides about 1 cm ventral to the position of the neurocentral sutures. There is no ridge on the midline on the ventral surfaces of any of the three trunk vertebrae of individual “B;” however there is a recognizable, but poorly developed ridge along the midline of the ventral surface of the centrum of the trunk vertebra of individual “A,” which suggests that the trunk vertebra of individual “A” is located more anteriorly in the column than the other trunk vertebrae.

All four of the trunk vertebrae preserve one or both of the accessory articulation structures (i.e., hyposphene, hypantrum) that form the hyposphene–hypantrum articulation. TMM 31025-1261.4 (Figs. 3A and 3C) is weathered so that a hyposphene could not be recognized, but a clear and deep hypantrum is visible on the anterior aspect of the neural arch. TMM 31025-1261.1 (Figs. 3B and 3D) is weathered so that a hypantrum could not be recognized, but on the posterior aspect of the neural arch there is a hyposphene preserved. TMM 31025-1261.2 (Figs. 3E and 3G) and TMM 31025-257 (Figs. 3F and 3H) preserve both hyposphenes and hypantra on their neural arches.

### Vertebrae (trunk; individual “A”)

#### TMM 31025-257

TMM 31025-257 is an anterior mid-trunk vertebra (Figs. 3F and 3H). The positions of the parapophyses and diapophyses place it as likely between presacral 12 and 14 (see above). The parapophysis is slightly more dorsally located on the neural arch than TMM 31025-1261.4. Both of the parapophyses and both the diapophyses are broken off at their bases. The neural spine, left and right postzygapophyses, and left prezygapophysis are broken off. The neural arch is firmly attached to the centrum, with a visibly raised evidence of where the neurocentral suture was. In anterior view, the neural canal is deeply excavated and elliptical as the dorsoventral axis is slightly elongated relative to the mediolateral axis.

The posterior and anterior neural arches of TMM 31025-257 preserve clearly developed hyposphene and hypantrum articulation structures, respectively. In posterior view, the hyposphene is broken, but its shape is identifiable; it is triangular with the ventral edge along the mediolateral horizontal plane and a point of the triangle directed dorsally, to where the articular surfaces of the postzygapophyses meet at ∼45° angles dorsal to the horizontal. The sides of the hyposphene each measure 1.2 cm and the base of the triangle is 0.8 cm across, although it is slightly arched as the dorsal boarder of the neural canal. The trunk vertebra of *P. kirkpatricki* (TTU P-9002, Weinbaum, 2013, Fig. 4), the trunk vertebra of *P. chiniquensis* (UFRGS-PV-0156-T), and the posterior trunk vertebra of *F. tenax* (PVL 3850, Bonaparte, 1981, Figs. 10 and 11) have similarly shaped hyposphenes in that they are triangular and connect with the postzygapophyses at the dorsally located point. *B. kupferzellensis* (SMNS 80296, Gower & Schoch, 2009, Fig. 2) has a hyposphene structure present on its trunk vertebrae that appears rectangular and dorsoventrally elongate. On TMM 31025-257, the face of the hyposphene is approximately 2 cm from the diapopohyses in lateral view. The hypantrum space between the prezygapophyses is 1 cm across. On the lateral edges of the hypantrum, centroprezygapophyseal laminae project laterally from the ventral side of the prezygapophyses, and they extend ventrally along the neural canal to the dorsal edge of the centrum.

The neural spine is laterally compressed, and roughly the same height as the height of the rest of the vertebra to the base of the neural spine. Grooves extend dorsoventrally along both the posterior and anterior surfaces of the neural spine. The spinoprezygapophyseal laminae are paired and cease to form distinct ridges about halfway up dorsoventrally from the base of the neural spine. The dorsal edge of the neural spine is rounded and the top centimeter of the neural spine is slightly laterally expanded, although it is not sufficiently wide to form a “spine table” as in the mid-trunk vertebrae of *N. songeaensis* (NMT RB48) and the anterior and posterior trunk vertebrae of *F. tenax* (PVL 3850). The neural spine of TMM 31025-257 is angled on the posterior edge and extends further dorsally on the anterior edge, causing it to be slightly wider at the distal end than at the base in lateral view, unlike the neural spines of the posterior trunk vertebrae of *P. kirkpatricki* (TTU P-9002) and *B. kupferzellensis* (SMNS 52970), which are both more rectangular in lateral view.

TMM 31025-257 has distinct laminae between the prezygapophyses, postzygapophyses, parapophyses, and diapophyses. On the lateral sides, the specimen has paradiapophyseal laminae, which connect the diapophyses with the parapophyses. There are anterior centroparapophyseal laminae, which connect the parapophyses with the anterior portion of the neurocentral junction, and there are also posterior centrodiapophyseal laminae, which connect the diapophyses with the posterior portion of the neurocentral junction, and all of these laminae are at a 45° angle ventral to the anteroposterior horizontal (Figs. 4B and 4D). The prezygapophyseal laminae are well defined and connect the parapophyses and the lateral aspect of the prezygapophyses at a 45° angle dorsal to the anteroposterior horizontal. The anterior centroparapophyseal laminae are recognizable but poorly defined, as the parapophyses are almost on the furthest anteroventral portion of the neural arch. The prezygadiapophyseal laminae are laterally thin and pronounced and connect the diapophyses and lateral edge of the prezygapophyses at a ∼10° angle to the anteroposterior horizontal with the articular surfaces of the prezygapophyses completely ventral to the diapophyses. TMM 31025-257 has centroprezygapophyseal laminae on each lateral side of the neural arch; they are thinner laterally in relation to the other laminae on this specimen’s neural arch, and both are vertically oriented. The postzygadiapophyseal laminae are thick in lateral view and poorly laterally expanded with the postzygapophyses and diapophyses only ∼0.5 cm apart (Figs. 4B and 4D). These laminae are at a ∼10° angle to the anteroposterior horizontal with the postzygapophyses being dorsal to the diapophyses. There are slight knobs extending along the anterodorsal portions of the postzygapophyses from the neural spine, but they do not appear pronounced enough for us to definitively call them epipophyses.

### Vertebrae (trunk; individual “B”)

#### TMM 31025-1261.4

TMM 30125-1261.4 is an anterior to mid-trunk vertebra. It is weathered post-depositionally, and the neural arch is broken horizontally through the mediolateral middle and separate completely from the centrum (Figs. 3A and 3C). It is further weathered so that the neural arch cannot be accurately reattached to the centrum in a close fit. Both postzygapophyses are broken off, and the neural spine is broken off near its base. This specimen can be attributed to presacral 11–13, as the parapophyses are well separated from the diapophyses, and the parapophyses are located at the junction of the neural arch and the centrum. The neural arch is fused to the centrum, and there is evidence of where a neurocentral suture was, which is slightly raised in some areas (Brochu, 1996).

On the left lateral side, TMM 30125-1261.4 has distinct laminae forming pronounced ridges, the posterior and anterior centrodiapophyseal laminae, which connect the diapophyses to the posterior and anterior portions of the neurocentral junction, respectively. The anterior centrodiapophyseal lamina is recognizable but not clearly defined on the right lateral side. On both lateral sides the prezygapophyseal laminae are recognizable and connect the parapophyses and the lateral aspect of the prezygapophyses at a steep angle (>80°) to the anteroposterior horizontal.

The posterior facet of the neural arch is sufficiently weathered so that no hyposphene structure is recognizable, however there is a well-defined hypantrum visible on the anterior facet of the neural arch (Fig. 3A and 3C). The hypantrum is 0.5 cm wide at its dorsal edge where it meets the articular faces of the prezygapophyses, and widens out ventrally to 1.1 cm at its ventral edge. The prezygapophyseal articular surfaces are gently curved and concave dorsally. They are positioned at ∼45° dorsal to the horizontal. The postzygapophyses are both completely broken off, and it is also unclear as to where they were originally positioned on the neural arch because of the extensive weathering. The neural spine is broken at 1.5 cm from its base. A deep groove extends dorsoventrally along what is preserved of the anterior surfaces of the neural spine. On either side of this groove, the spinoprezygapophyseal laminae are paired and form thin (<0.5 cm) and distinct ridges and are separated for the preserved length of the neural spine. On the right lateral side of the neural arch, in dorsal view, there is a deep fossa between the base of the neural spine and the prezygapophyseal lamina. The left lateral side is broken in this corresponding place, obstructing any potential lateral fossa.

#### TMM 31025-1261.1

TMM 31025-1261.1 is an anterior to mid-trunk vertebra that is nearly complete, but is well weathered post-depositionally, more so on the anterior and right lateral portions. The specimen is attributed to presacral 12–14, as the parapophyses are well separated from diapophyses, and both are located on the neural arch. The neural arch is completely fused to the centrum with only a slight textural trace of where the neurocentral suture was located (Brochu, 1996).

The parapophyses are slightly higher on the neural arch than those on the trunk vertebra we attribute to individual “B” (TMM 31025-257). Both of the parapophyses are broken off at their bases. They are circular in lateral view with a diameter of about 1 cm. The left parapophysis is in the correct anatomical position, but the right parapophysis has broken from its originally position and has shifted about 3–4 cm posteriorly. The right diapophysis is broken off at its base, and the left diapophysis is broken off about 1.5 cm from its base. This left diapophysis is dorsoventrally compressed, shaped as a narrow ellipse in lateral view with the long axis along the anteroposterior horizontal. There is a clear paradiapophyseal lamina connecting the parapophysis and the diapophysis on the left side. There is also a posterior centrodiapophyseal lamina connecting the diapophysis with the posterior section of the neurocentral suture junction. The right side is too weathered and broken to see any laminae. No zygapophyses are preserved. The neural canal is filled with sediment but appears to be only slightly laterally compressed (Figs. 3B and 3D).

The neural spine of TMM 31025-1261.1 is complete but is broken and offset dorsally by ∼25°. The neural spine is tall relative to the rest of the neural arch and laterally compressed and the dorsal edge of the neural spine is sloped at 45°, increasing in height posteriorly, in lateral view. The anteroposterior width of the neural spine is nearly uniform throughout in lateral view. The distal end of the neural spine is slightly rounded. The neural spine is posteriorly shifted in relation to the anteroposterior center of the centrum. The anterior surface of the neural arch is well weathered post-depositionally so that a hypantrum is not recognizable. The posterior surface of the neural arch preserves a clearly defined hyposphene. The shape of the hyposphene is triangular in posterior view, with a point directed dorsally along the dorsoventral midline, and the ventral side is slightly concave, forming the dorsal border of the neural canal (Figs. 3B and 3D). It has a similar shape to the hyposphene of TMM 31025-257, but the structure in TMM 31025-1261.1 extends much further distally from the neural arch. The sides of the hyposphene both measure 1 cm and the ventral edge, straight across from bottom of each side to the other and not along the concave surface, measures 1.2 cm. The connection between the hyposphene and the postzygapophyses cannot be seen, as the neural spine is broken and offset from the point just dorsal to the hyposphene and ventral to the articular surfaces of the postzygapophyses. There are clear spinoprezygapophyseal laminae and spinopostzygapophyseal laminae on the anterior and posterior aspects of the neural spine.

#### TMM 31025-1261.2

TMM 31025-1261.2 is a posterior trunk vertebra and we attribute it to presacral position somewhere among 14–17, as the parapophyses are only separated from the diapophyses by about 1 cm, and both are located on the neural arch. It is well weathered post-depositionally. The neural arch is completely fused to the centrum with slight bowing where the neurocentral suture was located, but no suture is present within the bone. The neural canal is deeply excavated and laterally compressed in anterior view, but appears round and less laterally compressed in posterior view (Figs. 3E and 3G).

On the lateral sides of this specimen, the diapophyses are broken about 2 cm from where they connect to the neural arch. Both are dorsoventrally compressed and thin out posteriorly from a circle with a diameter ∼1 cm across to a flat surface with a thickness of 0.5 cm. Both diapophyses are intact and both are dorsoventrally compressed so that they are shaped as a narrow elliptical with the long axis along the anteroposterior horizontal. There are clearly defined paradiapophyseal lamina connecting the parapophysis and the diapophysis on both lateral sides. There are also posterior centrodiapophyseal laminae connecting the diapophyses with the posterior sections of the neurocentral suture junction on both lateral sides.

The left postzygapophysis is broken off at its base, and the right postzygapophysis is broken off about 1.5 cm from its base and there is a subtle but recognizable postzygadiapophyseal lamina connecting the postzygapophysis to the diapophysis on the right lateral side. The right prezygapophysis is broken off at its base, and the left prezygapophysis is only broken slightly at the tip with no more than half a centimeter of missing material. There are clear prezygadiapophyseal laminae connecting the prezygapophyses with the diapophyses on both sides. There are also clear prezygaparapophyseal laminae connecting the prezygapophyses with the parapophyses on both sides.

The neural spine is broken off immediately dorsal to the prezygapophyses, so spinoprezygapophyseal laminae are obstructed from view. However, the break is not horizontal and there is more of the neural spine preserved in posterior view with the break on the posterior aspect of the neural spine located ∼3 cm dorsal to the postzygapophyses (Figs. 3E and 3G). There are clear spinopostzygapophyseal laminae extending dorsally on the intact portion of the neural spine from the postzygapophyses. There is a distinct hyposphene dorsal to where the bases of the postzygapophyses meet at a point. The shape of the hyposphene is triangular in posterior view, with a point directed dorsally, and the ventral side is slightly concave, curving around the dorsal portion of the neural canal (Figs. 3E and 3G). The hyposphene is similarly shaped to the hyposphene of TMM 31025-257; however it appears more dorsoventrally compressed in posterior view, and this does not appear to be related to compression of the fossil post-depositionally, as no other aspects of the vertebra appear more compressed. The sides of the hyposphene both measure 1.2 cm and from the ventralmost portion of one lateral side to the other, measures 1.5 cm. Although the right prezygapophysis is broken off, there is a clear hypantrum visible, and the space would have been ∼1 cm between the two prezygapophyses.

#### Rib (TMM 31025-2160)

**Figure 5:**
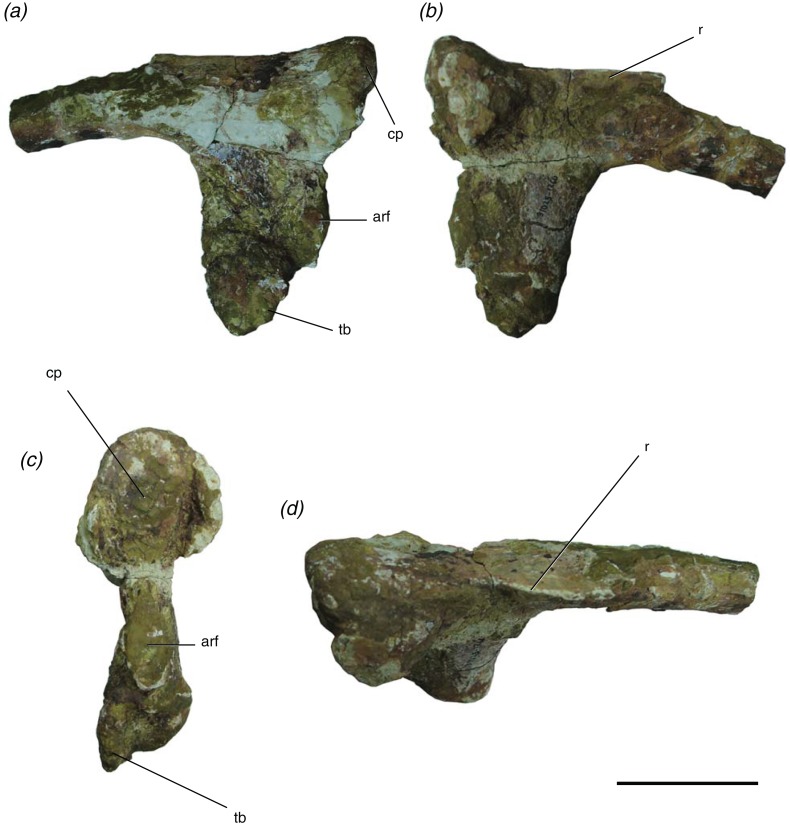
Presacral rib of *Poposaurus langstoni* (TMM 31025-2160) in (A, B) lateral views, (C) proximal view, and (D) dorsal view. Abbreviations: arf, accessory rib facet; cp, capitulum; r, ridge; tb, tuberculum. Scales = 5 cm.

This specimen is broken about 12 cm distally from the capitulum. The capitulum, tuberculum, and accessory rib facet are all intact. A thin ridge extends from the dorsal edge of the capitulum along the rib (Fig. 5). This rib fragment has an accessory articular facet that is located just dorsal to the tuberculum (Fig. 5). We assign it to the posterior cervical region of the axial skeleton because of the relative locations of the capitulum and tuberculum and the presence of an accessory rib facet, as a corresponding accessory rib articulation is seen on the posterior cervical vertebrae described in this paper (TMM 31025-177, TMM 31025-1262, TMM 31025-1261.3). An accessory rib facet between the capitulum and tuberculum is also present in *P. gracilis* (Weinbaum & Hungerbühler, 2007) and *A. babbitti* (Nesbitt, 2005), and it matches in position, just dorsal to the parapophysis to the accessory rib articulation structures in the posterior cervical vertebrae described herein. Weinbaum & Hungerbühler (2007) cited this accessory articulation as a synapomorphy of the clade Poposauroidea, which includes *P. gracilis*, *P. langstoni*, *E. okeeffeae*, and *A. babbitti*.

### Variation in the hyposphene–hypantrum articulation in *P. langstoni*

The hyposphene–hypantrum articulation of the trunk vertebrae of *P. langstoni* presents an important opportunity to examine within-vertebral column variation because these vertebrae are preserved in three dimensions so that articulation surfaces and hyposphene–hypantrum are visible and easy to discern. Moving posteriorly along the vertebral column, the hyposphene–hypantrum articulation is present in the anteriormost trunk vertebrae of the individuals represented by presacral 11 in individual “A” (TMM 31025-1261.4) and presacral 12 of individual “B” (TMM 31025-257). The articulation is also present in presacral 12 in individual “A” (TMM 31025-1261.2). All three of the preserved hyposphenes are triangular in posterior view, and the ventralmost edges of the triangular hyposphenes are oriented horizontally with their apices pointed dorsally along the midline. The ventrolateral corners of the triangular hyposphenes curve slightly ventrally around the neural canal. The hyposphenes (TMM 31025-1261.1, TMM 31025-1261.2, TMM 31025-257) appear to be more dorsoventrally compressed (i.e., smaller height to width ratio) in individual “B” than in individual “A.” This material described herein includes the twelfth presacral from both individuals (TMM 31025-1261.1, TMM 31025-257), so it is likely that this variation is not based on location in the vertebral column. The hypantra (TMM 31025-1261.4, TMM 31025-1261.2, TMM 31025-257) reflect a complementary shape of the hyposphene structures on their respective vertebrae. The horizontal distance between the prezygapophyses is narrower across the structure at the dorsal most edge than at the ventralmost extent, creating a triangular space for articulation. In summary, the hyposphene–hypantrum articulation appears triangular in all vertebrae described herein and therefore does not vary in general shape along the vertebral column of one individual of *P. langstoni*, but the two recognized individuals of *P. langstoni* have hyposphene structures that are markedly different in terms of height to width ratio (Table 1).

**Table 1:** Height and width of each of the hyposphenes preserved in this material.

Specimen #	Individual	Hyposphene dimensions		Height: width
		Height (mm)	Width (mm)	
TMM 31025-257	A	11	7	1.571428571
TMM 31025-1261.1	B	10	12	0.833333333
TMM 31025-1261.2	B	10	11	0.909090909

## Discussion

### Definition of the hyposphene–hypantrum articulation

The hyposphene–hypantrum articulation appears in many clades within Archosauria and has been defined simply as a vertical wall of bone ventral to the postzygapophyses and a notch between the prezygapophyses (Rauhut, 2003; Apesteguia, 2005; Hibbard & Williston, 1971). Those previous studies included definitions of the hyposphene–hypantrum articulation that were based on the vertebral morphology of saurischian dinosaurs, but they are not comprehensive in incorporating the variation in shape in dinosaurs and pseudosuchians. Furthermore, a number of pseudosuchians also have been reported to bear the hyposphene–hypantrum articulation without an explanation of why it is homologous with those structures in saurischian dinosaurs (Bonaparte, 1981; Weinbaum & Hungerbühler, 2007; Peyer et al., 2008; Gower & Schoch, 2009; Lautenschlager & Desojo, 2011; Weinbaum, 2013). This is important to clarify because the hyposphene–hypantrum has been cited as a synapomorphy of Saurischia (Gauthier, 1986) and of at least one clade of pseudosuchians (*Ticinosuchus* + Paracrocodylomorpha, Nesbitt, 2011). Here we apply an explicit definition of this articulation to facilitate identification and future studies of intervertebral articulation in archosaurs.

**Figure 6:**
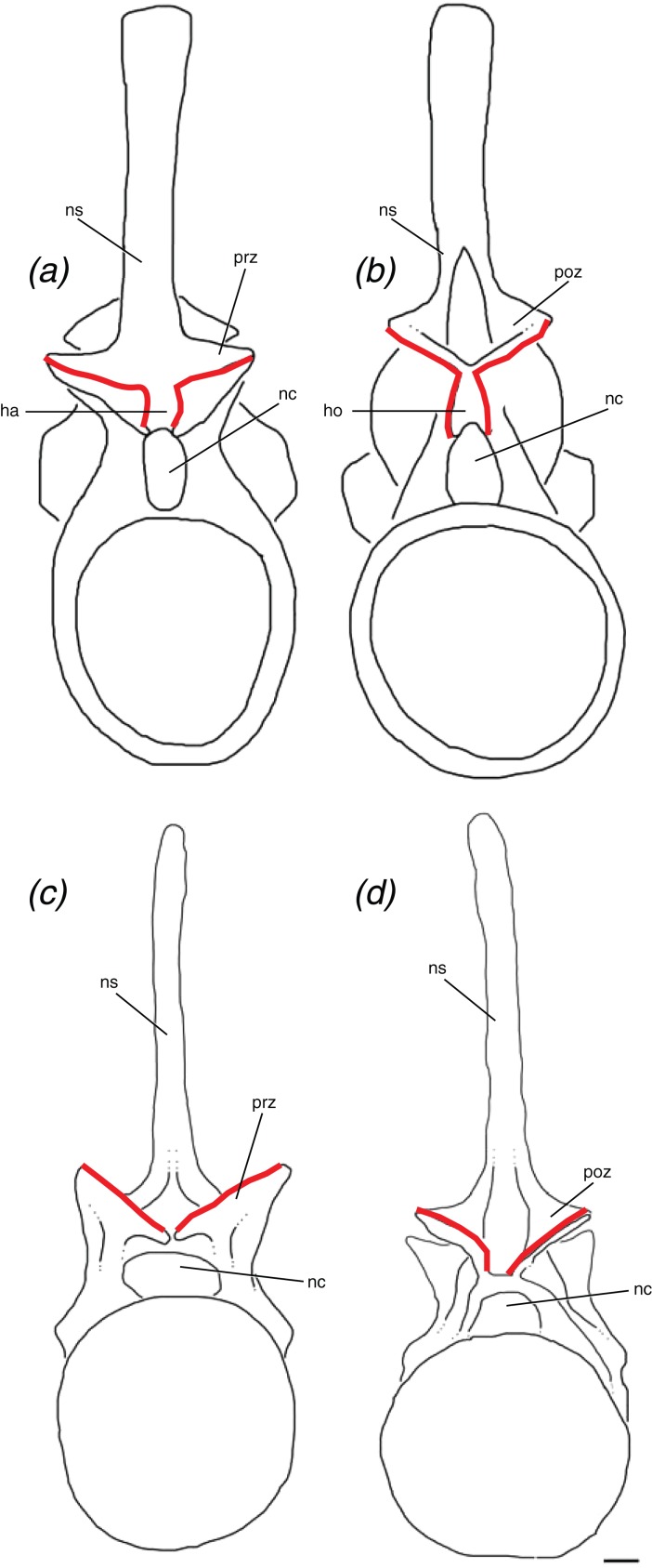
Schematic of two idealized archosaur vertebrae both with (A, B) modeled on *Poposaurus langstoni*, TMM 31025-257, and without (C, D) modeled on an unnamed phytosaur trunk vertebra, PEFO 26695 the hyposphene-hypantrum articulation in anterior (A, C) and posterior (B, D) views. Abbreviations: ha, hypantrum; ho, hyposphene; nc, neural canal; ns, neural spine; poz, postzygapophysis; prz, prezygapophysis. Scales = 5 cm.

**Figure 7:**
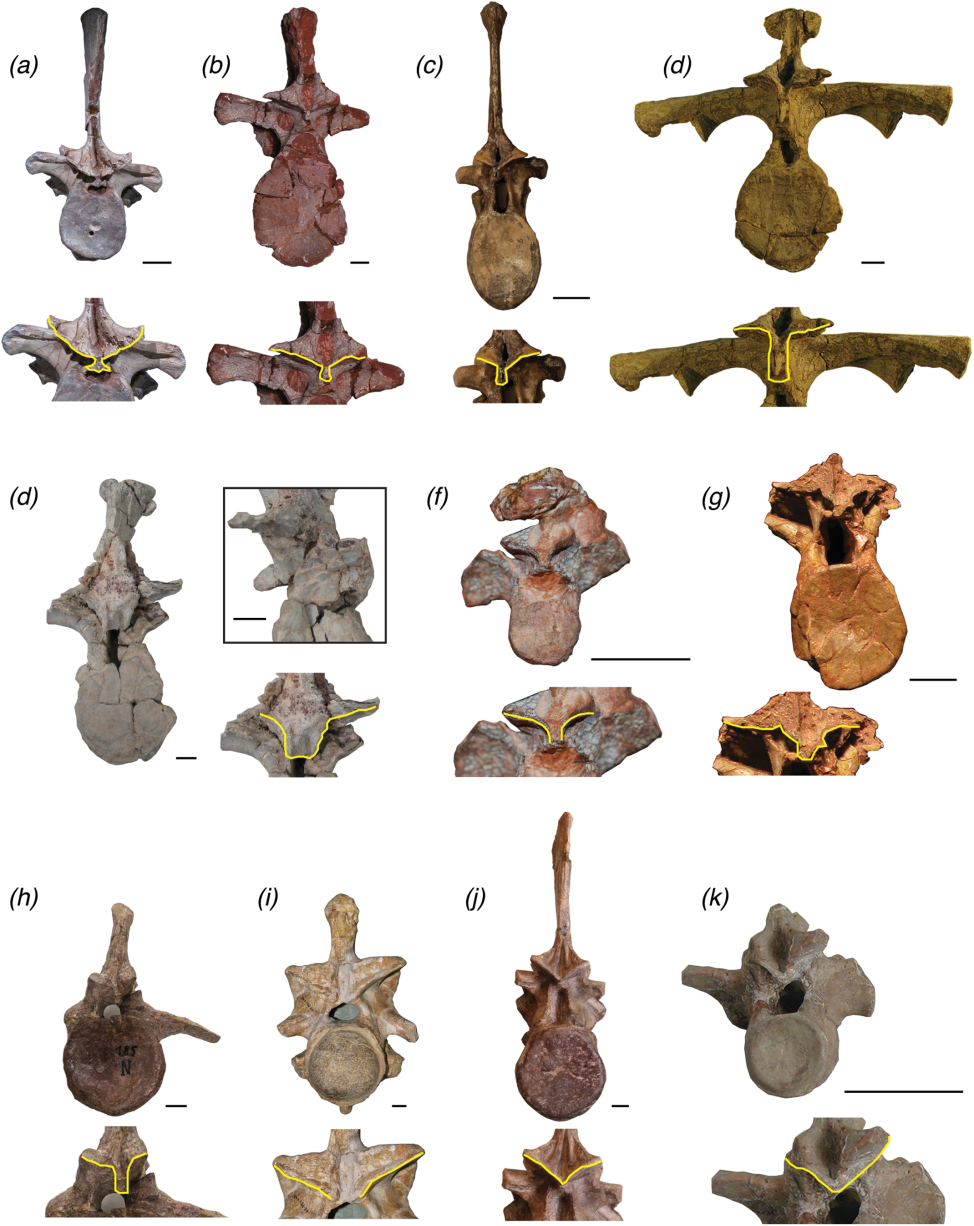
Trunk vertebrae of pseudosuchian archosaurs and closely related taxa in posterior view, showing examples of vertebrae with a hyposphene (A) *Postosuchus alisonae*, UNC 15575; (B) *Fasolasuchus tenax*, PVL 3850; (C) *Batrachotomus kupferzellensis*, SMNS 80296; (D) *Desmatosuchus spurensis*, MNA V9300; (E) *Scutarx deltatylus*, PEFO 31217; (F) *Aetobarbakinoides brasiliensis*, CPE2 168; (G) *Longosuchus meadei*,TMM 31100-148; (H) *Stagonosuchus nyassicus*, GPIT/RE/3832 and without a hyposphene (I) *Deinosuchus riograndensis*, TMM 43632-1; (J) *Erythrosuchus africanus*; (K) *Revueltosaurus callenderi*, PEFO 34561. Scales = 1 cm.

We define the hyposphene–hypantrum articulation as a bony projection, the hyposphene, on the posterior portion of the vertebra that fits into a complementary space, the hypantrum, on the anterior portion of the subsequent vertebra within a vertebral series (Fig. 6). Specifically, the hyposphene is located ventral to the articular surfaces of the postzygapophyses and is connected to these articular surfaces where they converge. It is located dorsal to the neural canal and in posterior view is symmetrical or nearly symmetrical across the midline. There is a distinct angle change (typically between ∼45° and 90°) between the articular surfaces of the postzygapophyses and the lateral surfaces of the hyposphene. The hyposphene projection must be a comparable shape and size to that of the hypantrum space of the subsequent vertebrae because these structures articulate precisely. The shapes in lateral view of the hyposphenes may appear as circular, square, dorsoventrally elongate rectangular, triangular, diamond, and quadrilateral, and the most common shapes found in pseudosuchian archosaurs are triangles and dorsoventrally elongate rectangles (Fig. 7). Though these shapes can vary between taxa, they also can vary within an individual. *P. langstoni* does not show drastic variation in hyposphene shape, but the proportions do vary along the column and between our two individuals (i.e., ratio of height to width increasing posteriorly in individual “B” and greater ratio of height to width in individual “A” than in individual “B”) (Table 1). As long as these projections extend posteriorly from the neural arch, their lateral surfaces are confluent with the articular surfaces of the postzygapophyses, and there is a distinct angle change between the articular surfaces of the postzygapophyses and the projection, any of these aforementioned shapes may be considered hyposphenes. Additionally, it is important to note that these structures are currently only known from trunk vertebrae posterior to the first nine presacral vertebrae.

Descriptions of a vertebra with the hyposphene–hypantrum should describe the shape that the hyposphene appears to be in posterior view. In vertebrae without the hyposphene, the articular surfaces of the postzygapophyses may converge but not form a ventrally elongated bony projection, as in *R. callenderi* (PEFO 34561) (Fig. 7K), *Erythrosuchus africanus* (SAM 905) (Fig. 7J), *P. gracilis* (NMT RB426), or *N. songeaensis* (NMT RB48), or they may not converge at all and appear well separated, as in *Deinosuchus riograndensis* (TMM 43632-1) (Fig. 7I) or *A. mississippiensis* (Romer, 1956, Fig. 130).

The lateral surfaces of the hyposphene articulate with the medial surfaces of the hypantrum, which is located between and ventral to the prezygapophyses and dorsal to the neural canal (Fig. 6). The articular surfaces of the prezygapophyses continue ventrally from their medial surfaces to form the articular surfaces of the hyposphene. As such, there must be a distinct angle change (typically between ∼45° and 90°) between these articular surfaces. In dorsal view, a hypantrum appears as a gap framed by parallel to sub-parallel medial surfaces of the prezygapophyses, which contact the neural arch just dorsal to the neural canal. In vertebrae without a hypantrum articulation (e.g., *R. callenderi*, PEFO 34561; *P. gracilis*, NMT RB426; *E. okeeffeae*, AMNH FR 30587; *D. riograndensis*, TMM 43632-1; *A. mississippiensis*, Romer, 1956), the medial edges of the prezygapophyses will converge in a “v” shape and will not appear parallel in dorsal view.

### Accessory intervertebral articulations within Pseudosuchia

The presence of the hyposphene–hypantrum articulation in *P. langstoni* allows for comparisons with the presacral vertebrae of other pseudosuchian archosaurs. Other closely related members of Poposauroidea, *P. gracilis*—the sister taxon to *P. langstoni*—and the smaller *E. okeeffeae*, have also been reported to have a hyposphene–hypantrum between trunk vertebrae (Weinbaum & Hungerbühler, 2007; Nesbitt, 2007).

The presacral vertebrae of the poposauroid *E. okeeffeae* (AMNH FR 30587, Nesbitt, 2007, Fig. 30) are only known from four semi-articulated vertebrae, and the anterior aspect of the neural arch is only visible on the anteriormost vertebra of the articulated series. There is not a clearly defined space between the prezygapophyses in this vertebra to satisfy our definition of a hypantrum. There is a slight gap between the medial aspects of the prezygapophyses, the articular surfaces of which are oriented horizontally, however these articular surfaces do not extend ventrally to form a hypantrum in accordance with our definition. No posterior surface of any vertebra of AMNH FR 30587 is clearly visible and intact, so we cannot conclusively state whether a hyposphene was present in the trunk vertebrae of known material of *E. okeeffeae*. Additionally, because we do not see a hypantrum present in this specimen of *E. okeeffeae*, we conclude that this taxon probably did not possess the hyposphene–hypantrum. Furthermore, there are no specimens of *Shuvosaurus inexpectatus* (e.g., TTU P-9001), the current sister taxon of *E. okeeffeae* (Nesbitt & Norell, 2006; Nesbitt, 2007, 2011), that preserve a neural arch where we could evaluate whether the hyposphene–hypantrum articulation was present or absent. The other known shuvosaurid, *S. longicervix* (PVSJ 85), is poorly preserved (Alcober & Parrish, 1997), and therefore it is difficult to determine definitively whether a hyposphene–hypantrum was present in that taxon as well. The sail-backed poposauroid *Lotosaurus adentus* (IVPP V 4880, IVPP V 48013) has not been reported to have the hyposphene–hypantrum articulation.

*Arizonasaurus babbitti* is one of the oldest members of Poposauroidea, outside of *L. adentus* + Shuvosauridae, and it has been reported to have the hyposphene–hypantrum articulation (MSM 4590, Nesbitt, 2005, 2007). The hyposphene structure of the trunk vertebra of *A. babbitti* (MSM 4590) is rectangular with the long axis oriented dorsoventrally in posterior view and located ventral to the postzygapophyses and dorsal to the neural canal (Nesbitt, 2005, Fig. 19). It does not have the triangular shape as in the trunk vertebrae of *P. langstoni*. The sister taxon of *A. babbitti* is the earliest diverging poposauroid, *Xilousuchus sapingensis* (IVPP V6026), and it has been cited as having the hyposphene–hypantrum as well (Nesbitt, Liu & Li, 2010). The ninth presacral vertebra figured by Nesbitt, Liu & Li (2010 Fig. 8) has a clearly defined hyposphene, and it is square in posterior view. A hypantrum between the prezygapophyses is clearly seen in anterior view and is of comparable size and shape to the hyposphene of the same vertebra (Nesbitt, Liu & Li, 2010, Fig. 8). The identification of this vertebra as the ninth presacral (Nesbitt, Liu & Li, 2010) may be incorrect given that there are no other taxa with a hyposphene in the first nine presacrals. Alternatively, the presence of a hyposphene in this position may be autapomorphic for this taxon.

**Figure 8:**
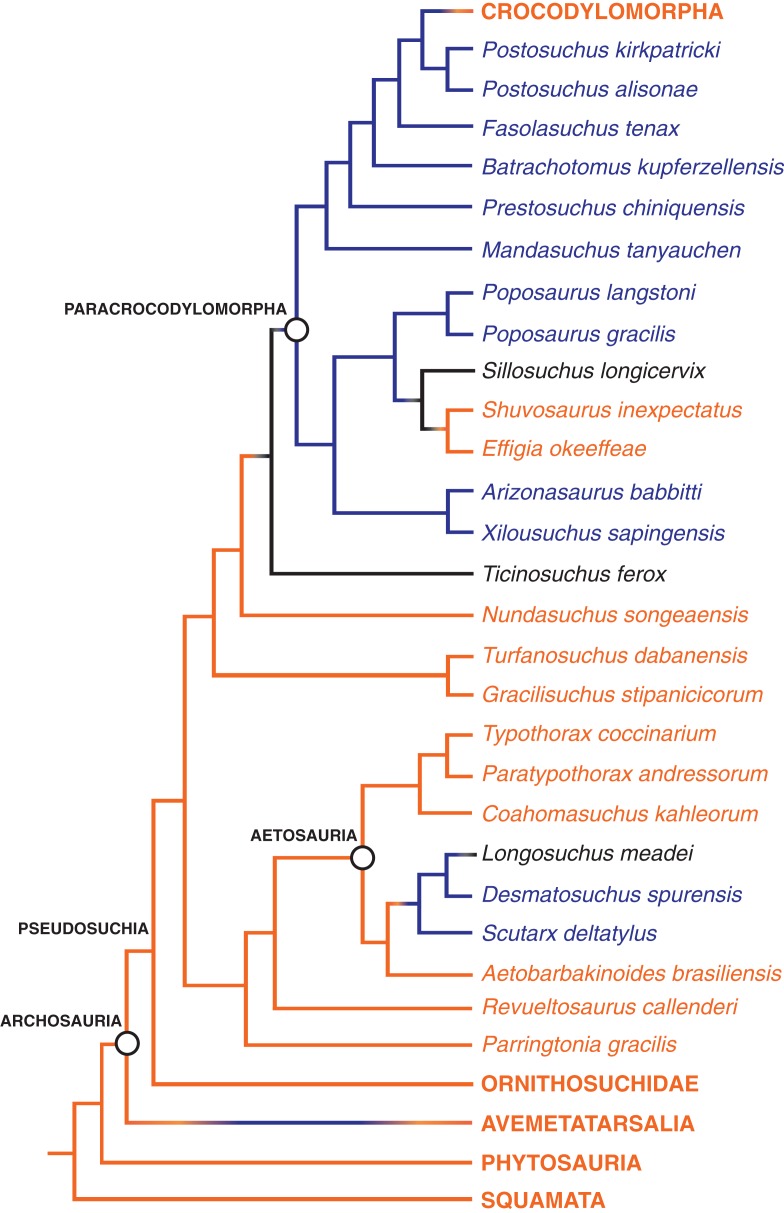
The distribution of the hyposphene–hypantrum articulation in Pseudosuchia and close relatives. Relationships from Nesbitt, Liu & Li (2010) and Nesbitt et al. (2014). Hyposphene–hypantrum present = blue; hyposphene–hypantrum absent = orange; ambiguous for hyposphene–hypantrum presence/absence = black.

Several Triassic loricatan paracrocodylomorphs and their closest relatives possess the hyposphene–hypantrum articulation, and this structure varies in shape and structure between taxa and even within an individual. *Stagonosuchus nyassicus* (GPIT/RE/3831, von Huene, 1938; Gebauer, 2004; Lautenschlager & Desojo, 2011) is reported to have the hyposphene–hypantrum in the trunk vertebrae. The anterior trunk vertebra of *S. nyassicus* (GPIT/RE/3831-9, von Huene, 1938; Gebauer, 2004; Lautenschlager & Desojo, 2011, Fig. 7) has a clearly defined hyposphene that is rectangular, but it is only slightly elongated dorsoventrally. The posterior trunk vertebra of *S. nyassicus* (GPIT/RE/3831-14, von Huene, 1938; Gebauer, 2004; Lautenschlager & Desojo, 2011, Fig. 7) has a hyposphene that is narrower mediolaterally and more elongated dorsoventrally than the hyposphene of its anterior trunk vertebra. This shape is distinctly different from the triangular hyposphenes of *P. langstoni* (Fig. 3). In anterior view, there is a clear space between the prezygapophyses to form a hypantrum in *S. nyassicus. T. ferox* is a close relative to *S. nyassicus* (Lautenschlager & Desojo, 2011), but the presence of the hyposphene–hypantrum articulation in that taxon is ambiguous because the known material (PIZ T 2817) is preserved in a flattened slab, and the vertebral column is mostly articulated so that the anterior and posterior aspects of the vertebrae cannot clearly be seen.

*Fasolasuchus tenax* (PVL 3850, Bonaparte, 1981, Figs. 10 and 11), *B. kupferzellensis* (SMNS 80296, Gower & Schoch, 2009, Fig. 2), *P. chiniquensis* (UFRGS-PV-0156-T, Azevedo, 1991, Fig. 11), and *Saurosuchus galilei* (PVSJ 32, Trotteyn, Desojo & Alcober, 2011, Fig. 7) have hyposphene–hypantrum articulations present in their trunk vertebrae. *F. tenax* has a hyposphene structure that appears rectangular in posterior view in the anterior trunk vertebra figured by Bonaparte (1981, Fig. 10). In the posterior trunk vertebra of *F. tenax* (Fig. 7B), the hyposphene structure is triangular in posterior view with the midline apex directed dorsally and the ventrolateral corners curved slightly ventrally around the neural canal, similar to that of *P. langstoni* and *P. kirkpatricki* (TTU P-9002). The trunk vertebra of *B. kupferzellensis* figured in lateral view by Gower & Schoch (2009, Fig. 2) (SMNS 80296) has a clearly defined hyposphene that is rectangular and elongated dorsoventrally in posterior view (Fig. 7C); in anterior view there is a clear space between the prezygapophyses to form a hypantrum in this specimen. The posterior trunk vertebra of *P. chiniquensis* that was figured in posterior view by Azevedo (1991, Fig. 11) has a well-defined hyposphene that is rectangular with the long axis oriented dorsoventrally in posterior view. The shape of this hyposphene most closely resembles the hyposphene shapes in *B. kupferzellensis* (SMNS 80296) (Fig. 7C) and *P. kirkpatricki* (TTU P-9002)*. S. galilei* (PVSJ 32) has a hyposphene–hypantrum articulation present on at least one of its posterior trunk vertebrae (Trotteyn, Desojo & Alcober, 2011), but it is difficult to tell the shape because of poor preservation of the specimen.

A large member of the paracrocodylomorph clade Rauisuchidae, *P. kirkpatricki* has been reported to have the hyposphene–hypantrum articulation present on its middle and posterior trunk vertebrae (TTU P-9002, Weinbaum, 2013). The figured anterior trunk vertebra and posterior trunk vertebra of TTU P-9002 have clearly defined hyposphene structures that appear triangular in posterior view, with the ventralmost aspect as a horizontal surface and the dorsalmost aspect as a midline apex (TTU P-9002, Weinbaum, 2013, Figs. 4 and 5). The ventrolateral corners of the triangular hyposphene also slightly curve around the neural canal at its dorsal margin. This shape is nearly identical to those hyposphenes of *P. langstoni* in the anterior trunk vertebrae TMM-31025-1261.1 and TMM-31025-1261.2. The hypantrum of the anterior portion of the neural arch of the anterior (Weinbaum, 2013, Fig. 4) and posterior (Weinbaum, 2013, Fig. 5) trunk vertebrae of *P. kirkpatricki* (TTU P-9002) also appear triangular in anterior view with a shape and size complementary to the hyposphene of the same vertebra. The hyposphene structure of the mid-trunk vertebra of *P. kirkpatricki* (TTU P-9002, Weinbaum, 2013, Fig. 4) appears rectangular in posterior view with the long axis oriented dorsoventrally. The one known trunk vertebra of *P. alisonae* (UNC 15575) has clear hyposphene and hypantrum structures (Fig. 7A). The hyposphene appears triangular in posterior view, similar to those of the anterior and posterior trunk vertebrae of *P. kirkpatricki* (TTU P-9002), with the midline apex directed dorsally and the ventrolateral corners curved slightly around the dorsal edge of the neural arch (Peyer et al., 2008, Fig. 4). The hypantrum of *P. alisonae* is of complementary shape and size to the hyposphene.

Just outside of Paracrocodylomorpha, *N. songeaensis* (NMT RB48, Nesbitt et al., 2014) was reported to possess the hyposphene–hypantrum articulation on its mid-trunk vertebrae. Evidence for a hypantrum was cited as a small gap between the anterior portion of the prezygapophyses, and the hyposphene was cited as a thin, ventrally directed lamina of bone between the postzygapophyses. However, this proposed hyposphene was not clearly defined, and appears simply as the postzygapophyses converging at the midline, without an extension of a bony process ventral to them (NMT RB48, Nesbitt et al., 2014, Fig. 4). There is a slight gap between the medial aspects of the prezygapophyses; however, articular surfaces do not extend ventrally from the prezygapophyses to form a hypantrum in accordance with our refined definition. This morphology in *N. songeaensis* is similar to that in *E. okeeffeae*, which we have concluded does not have the hyposphene–hypantrum articulation.

Hyposphene–hypantrum articulations have been reported in some members of Aetosauria, a group of quadrupeds with an extensive osteoderm carapace (Parker, 2008; Desojo, Ezcurra & Kischlat, 2012; Parker, 2016b). The largest known aetosaur, *D. spurensis* (MNA V9300, Parker, 2008), has hyposphene–hypantrum articulations between its trunk vertebrae. The hyposphene structures of the anterior and mid-trunk vertebrae are rectangular in posterior view and more elongated dorsoventrally relative to the hyposphene structures of the posterior trunk vertebrae (Fig. 7D). The rectangular hyposphenes of the anterior and mid-trunk vertebrae of *D. spurensis* appear more similar to those structures of *B. kupferzellensis*, *F. tenax*, and *P. chiniquensis* than to taxa with triangular hyposphenes (e.g., *P. langstoni*). The posterior trunk vertebrae of *D. spurensis* have hyposphenes that have a width and height nearly equal to each other. These hyposphenes also curve around the neural canal and appear triangular in posterior view (MNA V9300, Parker, 2008, Fig. 9). These triangular hyposphenes of the posterior trunk vertebrae appear more similar to the structures of the anterior trunk vertebrae of *P. langstoni* and *P. kirkpatricki* than to taxa with rectangular hyposphenes.

**Figure 9:**
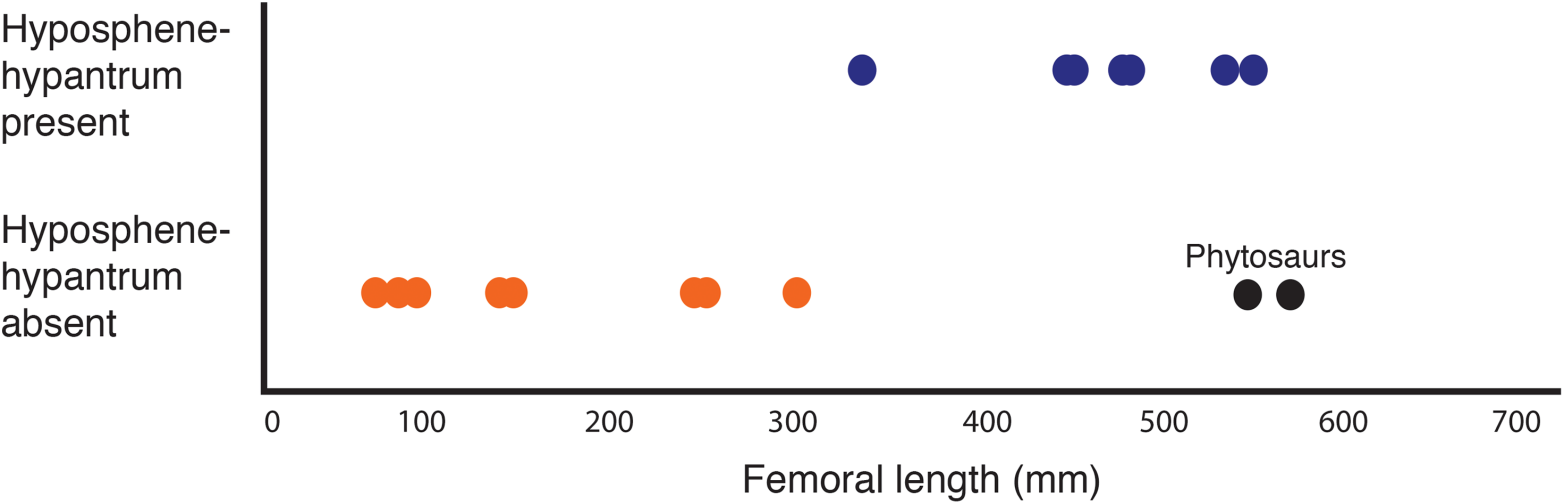
Plot showing femoral length versus hyposphene–hypantrum presence or absence (Table 2) with pseudosuchian archosaurs as data points (*n* = 24). Hyposphene–hypantrum present = blue; hyposphene–hypantrum absent = orange. Each data point corresponds to a taxon included in Table 2. See Table 2 caption for explanation about color and placement of the two phytosaur data points.

Several other aetosaurs besides *D. spurensis* have been reported to have the hyposphene–hypantrum articulation including *S. deltatylus* (PEFO 34045, Parker, 2016b), possibly *Longosuchus meadei* (TMM 31100-448, TMM 31100-452), and *Aetobarbakinoides brasiliensis* (CPE 2 168, Desojo, Ezcurra & Kischlat, 2012). However, a hyposphene structure is difficult to infer on most of the vertebrae of *A. brasiliensis* (CPE 2186, Desojo, Ezcurra & Kischlat, 2012, Fig. 5) (Fig. 7F) because much of the holotype is articulated or broken on the neural arch. Desojo, Ezcurra & Kischlat (2012) described *A. brasiliensis* as having hyposphenes that are “Y-shaped” where the elongated hyposphene structure forms the “trunk” of the “Y” and the postzygapophyses connected and dorsal to it form the top two dorsolaterally projecting “branches” of the “Y” in posterior view. On our close inspection of the well-preserved and non-articulated vertebrae of *A. brasiliensis*, there does not appear to be a distinct angle change between the articular surfaces of the postzygapophyses to the bony projection ventral to and between them (Fig. 7F). Therefore we conclude that *A. brasiliensis* does not have a hyposphene–hypantrum according to our definition. Parker (2016b) did not consider the aetosaur *S. deltatylus* to have a true hyposphene–hypantrum, however the “ventral bar” structure on the posterior aspect of its vertebrae (Parker, 2016b, Fig. 12) does fit our definition of a true hyposphene. *S. deltatylus* has two different shape varieties of hyposphene structures present in its trunk vertebrae: one that is circular in posterior view and tapers to a point distally from the body of the vertebra (PEFO 34045 FF-22, Parker, 2016b, Fig. 13) (Fig. 7E) and a second that is triangular with a dorsally oriented point extending along the dorsoventral margin of the neural arch (PEFO 34045 FF-51, Parker, 2016b, Fig. 12). *S. deltatylus* also preserves hypantrum structures of complementary size and shapes on the anterior aspects of its neural arches. *Scutarx robertsoni* (Walker, 1961, Fig. 7) has a “ventral bar” shaped hyposphene in an anterior dorsal vertebra (R 4799) that appears very similar to that of *S. deltatylus. S. robertsoni* also preserves complementary hypantrum structures on the anterior aspects of the anterior dorsal (R 4799) and posterior cervical (E.M. 30R) vertebrae (Walker, 1961, Fig. 7).

The aetosaur *L. meadei* (TMM 31100-448, TMM 31100-452) may have a hyposphene–hypantrum articulation in its trunk vertebrae, but this is ambiguous because of the poor preservation of known material. The trunk vertebra of *L. meadei*, TMM 31100-448 appears to have a hyposphene that is square in posterior view; however, the postzygapophyses and surrounding portions of the neural arch are broken and compressed, making it difficult to definitively discern a hyposphene structure according to our definition (Fig. 7G). Neither *Typothorax coccinarium* (NMMNH P-12964, Heckert et al., 2010, Fig. 4A) nor *Paratypothorax andressorum* (PEFO 3004, Lucas, Heckert & Rinehart, 2006; Hunt & Lucas, 1992, Fig. 4) was reported to have the hyposphene–hypantrum, and we concur with this through our observations of the published figures of those materials and through direct observation. The small aetosaur *Coahomasuchus kahleorum* (TMM 31100-437, Parker, 2016a) does not appear to have the hyposphene–hypantrum in its trunk vertebrae; however, the known vertebral material is mostly articulated, poorly preserved, and portions of the neural arches are broken or obscured (TMM 31100-437).

Although it is present in a few aetosaurs, the hyposphene–hypantrum articulation is not present in the sister taxon to Aetosauria, *R. callenderi* (PEFO 34561) (Fig. 7K). It is also not present in *P. gracilis* (NMT RB426), the sister taxon of *R. callenderi* (PEFO 34561) + Aetosauria (Nesbitt et al., in press). The postzygapophyses of the vertebrae of *P. gracilis* converge at the midline but they do not extend into a projection ventrally. The articular surfaces of the postzygapophyses of the few known anterior trunk vertebrae of *R. callenderi* that are intact and not obscured by sediment (PEFO 34561-DVf, PEFO 34561-DVh) do converge; however, they do not have a bony and dorsoventrally elongated process that could definitively be a hyposphene. The cervical vertebrae (PEFO 34561-CV) of this specimen definitively do not have the hyposphene–hypantrum articulation. Therefore, both *R. callenderi* and *P. gracilis* are small pseudosuchian archosaurs that lack the hyposphene–hypantrum.

### Relationship of the hyposphene–hypantrum articulation with body size

The articulation structures of the hyposphene–hypantrum are widespread in the clade Pseudosuchia, but are noticeably absent in the group Crocodylomorpha (e.g., *Sphenosuchus acutus,* UCMP 129740; *D. riograndensis*, TMM 43632-1; *A. mississippiensis*, TMM M-12606) (Fig. 8), which includes the only living pseudosuchians, the crocodylians. Most early members of Crocodylomorpha are markedly smaller (i.e., shorter femoral lengths) (Turner & Nesbitt, 2013) than their paracrocodylomorph relatives (Fig. 9), such as the poposaurids (e.g., *P. langstoni*; *P. gracilis*, Weinbaum & Hungerbühler, 2007, TTU P-10419), the rauisuchid *P. kirkpatricki* (TTU-P 9002, Weinbaum, 2013), other pseudosuchians (i.e., *P. chiniquensis*, UFRGS-PV-0156-T, Azevedo, 1991; *F. tenax*, PVL 3850, Bonaparte, 1981; *S. nyassicus*, GPIT/RE/3831, Lautenschlager & Desojo, 2011; *Mandasuchus tanyauchen*, NHMUK PV R6792, Butler et al., in press), and the largest-known aetosaur *D. spurensis* (MNA V9300, Parker, 2008). Because the hyposphene–hypantrum articulation is in many large-bodied taxa but not in smaller ones (Fig. 9), we explored this potential correlation with body size by assigning each taxon we examined (i.e., each taxon included in our phylogenetic tree, Fig. 8) to either presence or absence of the hyposphene–hypantrum (Table 2), and then plotted these two groups by their femoral length as an estimate for body size (see methods in Fariña, Vizcaíno & Bargo, 1998; Christiansen & Fariña, 2004; Farlow et al., 2005; Carrano, 2006; Turner & Nesbitt, 2013). With these data, we found a relationship between larger body sizes and the presence of the hyposphene–hypantrum articulation: taxa with femora length >300 mm typically have this extra articulation (Fig. 9). While our data support this correlation, the relationship is solely visual at present and deserves rigorous statistical testing, which will be done in future studies.

**Table 2:** Table listing all taxa used in Fig. 8, with the addition of two phytosaurs (*Smilosuchus gregorii*, *Machaeroprosopus pristinus*), and includes whether we consider the taxon to possess a true hyposphene–hypantrum articulation, the taxon’s femoral length, and the source(s) we used for the femoral length data.

Taxon	Hyposphene–hypantrum? (Y/N)	Femoral length (mm)	Source
*Postosuchus kirkpatricki*	Y	528	Turner & Nesbitt (2013)
*Postosuchus alisonae*	Y	558	Turner & Nesbitt (2013)
*Fasolasuchus tenax*	Y	750	Turner & Nesbitt (2013)
*Batrachotomus kupferzellensis*	Y	420	Turner & Nesbitt (2013)
*Prestosuchus chiniquensis*	Y	538	Turner & Nesbitt (2013)
*Mandasuchus tanyauchen*	Y	212	Butler et al. (in press)
*Poposaurus langstoni*	Y	353	Turner & Nesbitt (2013)
*Poposaurus gracilis*	Y	353	Turner & Nesbitt (2013)
*Sillosuchus longicervix*	?	440	Alcober & Parrish (1997)
*Shuvosaurus inexpectatus*	N	255	Turner & Nesbitt (2013)
*Effigia okeeffeae*	N	301	Turner & Nesbitt (2013)
*Arizonasaurus babbitti*	Y	490	Turner & Nesbitt (2013)
*Xilousuchus sapingensis*	Y	302	Turner & Nesbitt (2013)
*Ticinosuchus ferox*	?	240	Turner & Nesbitt (2013)
*Nundasuchus songeaensis*	N	230	NMT RB48
*Turfanosuchus dabanensis*	N	136	Turner & Nesbitt (2013)
*Gracilisuchus stipanicicorum*	N	78	Turner & Nesbitt (2013)
*Typothorax coccinarium*	N	291.8	Heckert et al. (2010)
*Paratypothorax andressorum*	N	?	Hunt & Lucas (1992) and Lucas, Heckert & Rinehart (2006)
*Coahomasuchus kahleorum*	N	109	Kubo & Kubo (2012) and Parker (2016a)
*Longosuchus meadei*	?	337	Turner & Nesbitt (2013)
*Desmatosuchus spurensis*	Y	450	Parker (2008)
*Scutarx deltatylus*	Y	?	Parker (2016b)
*Aetobarbakinoides brasiliensis*	N	120	Desojo, Ezcurra & Kischlat (2012)
*Revueltosaurus callenderi*	N	90	PEFO 34561
*Parringtonia gracilis*	N	74	NMT RB426
*Smilosuchus gregorii*	N	545	Turner & Nesbitt (2013)
*Machaeroprosopus pristinus*	N	444	Turner & Nesbitt (2013)

**Notes:**

The colored rows correspond to the data points used in Fig. 9, with the colors corresponding to presence/absence of the hyposphene–hypantrum (blue = present, orange = absent). The black rows are taxa that are ambiguous for either presence/absence or lack femoral length data, and these taxa are therefore not included in Fig. 9. The phytosaurs are also represented in black in this table because of the ambiguous phylogenetic position of Phytosauria as either early diverging within Pseudosuchia or as the sister group to Pseudosuchia. Because of their lack of a true hyposphene–hypantrum, they are depicted in Fig. 9 under that classification in the color black.

An exception to this body size relationship is phytosaurs, which are either the earliest diverging pseudosuchians (Gauthier, 1986; Benton & Clark, 1988; Sereno, 1991; Benton, 1999; Ezcurra, 2016) or the sister group of Archosauria (Nesbitt, 2011). All taxa in this clade that we observed lack the hyposphene–hypantrum articulation but some have femoral lengths greater than many pseudosuchians that possess the articulation (e.g., *Smilosuchus gregorii*, 545 mm; *Machaeroprosopus pristinus*, 444 mm) (Fig. 9). The absence of the hyposphene–hypantrum in phytosaurs may be related to their inferred semi-aquatic ecology (Parrish & Padian, 1986), which is divergent from the terrestrial ecology of other early pseudosuchians. This could mean that femoral length cannot explain the presence or absence of the hyposphene–hypantrum in aquatic forms, and this may also explain the absence of the structure in the transition to a more aquatic ecology within Crocodylomorpha.

In summary, our observations of pseudosuchian archosaurs both with and without the hyposphene–hypantrum articulation showed a clear relationship between larger body sizes and the presence of the hyposphene–hypantrum articulation in trunk vertebrae across pseudosuchian archosaurs. Because of this close fit, this feature may be controlled by increases or decreases in body size and not strictly by inheritance. If so, the presence or absence of hyposphene–hypantrum would have limited use as a character in phylogenetic analyses of archosaurs (Gauthier, 1986; Nesbitt, 2011) and the character should be applied with caution when used to assign isolated vertebrae to pseudosuchian clades.

## Institutional Abbreviations

AMNH: American Museum of Natural History, New York, NY, USA
GPIT: Institut für Geowissenschaften, Universität Tubingen, Germany
GR: Ruth Hall Museum of Paleontology, Ghost Ranch, NM, USA
IVPP: Institute of Vertebrate Paleontology and Paleoanthropology, Beijing, China
MNA: Museum of Northern Arizona, Flagstaff, AZ, USA
MSM: Arizona Museum of Natural History (formerly Mesa Southwest Museum), Mesa, AZ, USA
NMMNH: New Mexico Museum of Natural History and Science, Albuquerque, NM, USA
NMT: National Museum of Tanzania, Dar es Salaam, Tanzania
PEFO: Petrified Forest National Park, Petrified Forest, AZ, USA
PVL: Instituto Miguel Lillo, Tucumán, Argentina
PVSJ: Division of Paleontology of the Museo de Ciencias Naturales de la Universidad Nacional de San Juan, San Juan, Argentina
SAM: Iziko: South African Museum, Cape Town, South Africa
SMNS: Staatliches Museum für Naturkunde, Stuttgart, Germany
TMM: Jackson School of Geosciences Vertebrate Paleontology Laboratory, University of Texas at Austin, Austin, TX, USA
TTU P: Texas Tech University Museum, Lubbock, TX, USA
UCMP: University of California Museum of Paleontology, Berkeley, CA, USA
UFRGS-PV: Instituto de Geociencias, Universidade Federal do Rio Grande do Sul, Porto Allegre, Brazil
UNC: University of North Carolina, Chapel Hill, NC, USA
USNM: National Museum of Natural History, Washington, DC, USA

## Additional Information and Declarations

### Data Availability

The following information was supplied regarding data availability: Material is registered at TMM—Jackson School of Geosciences Vertebrate Paleontology Laboratory, University of Texas at Austin, Austin, TX, USA.
